# Virus‐induced phytohormone dynamics and their effects on plant–insect interactions

**DOI:** 10.1111/nph.17261

**Published:** 2021-03-19

**Authors:** Li‐Long Pan, Huiying Miao, Qiaomei Wang, Linda L. Walling, Shu‐Sheng Liu

**Affiliations:** ^1^ Ministry of Agriculture Key Laboratory of Molecular Biology of Crop Pathogens and Insects Institute of Insect Sciences Zhejiang University Hangzhou 310058 China; ^2^ Key Laboratory of Horticultural Plant Growth Development and Quality Improvement Ministry of Agriculture Department of Horticulture Zhejiang University Hangzhou 310058 China; ^3^ Department of Botany and Plant Sciences Center for Plant Cell Biology University of California Riverside, CA 92521‐0124 USA

**Keywords:** ethylene, insect vector, jasmonates, molecular mechanism, plant hormone, plant virus, salicylic acid, virus–plant–insect tripartite interactions

## Abstract

Attacks on plants by both viruses and their vectors is common in nature. Yet the dynamics of the plant–virus–vector tripartite system, in particular the effects of viral infection on plant–insect interactions, have only begun to emerge in the last decade. Viruses can modulate the interactions between insect vectors and plants via the jasmonate, salicylic acid and ethylene phytohormone pathways, resulting in changes in fitness and viral transmission capacity of their insect vectors. Virus infection of plants may also modulate other phytohormones, such as auxin, gibberellins, cytokinins, brassinosteroids and abscisic acid, with yet undefined consequences on plant–insect interactions. Moreover, virus infection in plants may incur changes to other plant traits, such as nutrition and secondary metabolites, that potentially contribute to virus‐associated, phytohormone‐mediated manipulation of plant–insect interactions. In this article, we review the research progress, discuss issues related to the complexity and variability of the viral modulation of plant interactions with insect vectors, and suggest future directions of research in this field.

1

**Table:** 

**Contents**		
	Summary	1305
I.	Introduction	1305
II.	Phytohormone‐mediated effects of virus infection on plant–insect interactions	1306
III.	Effects of virus infection on phytohormones other than JA, SA and ET	1308
IV.	Additional factors involved in virus‐induced changes of the tripartite interactions	1311
V.	Perspectives	1312
VI.	Prospects	1315
	Acknowledgements	1316
	References	1317

## I. Introduction

Plants, in both natural populations and cultivated fields, are constantly facing threats from multiple kinds of organisms, including pathogens and herbivores. Of the pathogens, plant viruses are obligate intracellular parasites that infect living plants and exclusively live and multiply within their host cells. Currently, they account for almost half of emerging infectious diseases of plants, seriously threatening the sustainability of crop production and, by extension, human survival (Anderson *et al*., 2004; Scholthof *et al*., 2011). Recent decades have seen an increase in plant viral diseases, for example the diseases caused by begomoviruses. This highlights the urgent need to advance our understanding of virus biology and, ultimately, the management of viral diseases (Navas‐Castillo *et al*., 2011).

With over half of the one million known insect species feeding on plants (Gangwere, 2008; Bernays, 2009), there are innumerable opportunities for novel insect, virus and plant interactions. Simultaneous occurrence of viral infection and insect infestation is common in plants (Stout *et al*., 2006; Wu & Baldwin, 2010; Mandadi & Scholthof, 2013). Thus, intensive interactions among viruses, their vectors and plants are inevitable. These relationships have important implications not only for individual organisms, but also for the population dynamics of each of the species involved in a given ecosystem (Stout *et al*., 2006; Tack & Dicke, 2013; Carr *et al*., 2018; Donnelly & Gilligan, 2020). Thus, detailed elucidation of the nature of these interactions will translate into improved knowledge of ecology, evolution and plant defense, thereby providing useful information for the development of novel control strategies to combat plant pathogens and insect pests.

A growing body of evidence shows that virus infection often exerts a significant influence on plant–insect interactions. As suggested by Mauck *et al*. (2012, 2018), the mode of transmission exerts substantial impact in shaping the pattern of effects of virus infection on plant–insect interactions. Viruses often manipulate plant–insect interactions to maximize their own spread. Specifically, for persistently transmitted viruses, acquisition and transmission of a virus require long‐term feeding by vectors on infected plants. These viruses tend to improve host suitability for vector feeding and consequently enhance vector populations. By contrast, nonpersistently transmitted viruses, which can be efficiently acquired and transmitted by insect vectors via brief probes, tend to reduce plant quality and promote dispersal of their vectors. Studies using mathematical modeling indicate that for nonpersistently transmitted viruses, virus‐induced inhibition or promotion of incessant insect vector feeding may facilitate virus spread at small and large spatial scales, respectively (Donnelly *et al*., 2019). While virus infections are now known to mediate multiple aspects of plant–insect interactions, detailed studies of the tripartite interactions between plants, viruses and insect vectors, especially the underlying behavioral, physiological and molecular mechanisms, have received wide attention from scientists only in the last decade. Case studies indicate that virus‐mediated changes of plant hormone pathways play a critical role in the tripartite interactions (Mauck *et al*., 2010; Thaler *et al*., 2010; Ziebell *et al*., 2011; Zhang *et al*., 2012; Casteel *et al*., 2014, 2015; Kersch‐Becker & Thaler, 2014; Li *et al*., 2014; Shi *et al*., 2016; Su *et al*., 2016; Wu *et al*., 2017; P. Li *et al*., 2019).

Phytohormones are organic chemicals that are produced endogenously and function as signals to coordinate plant growth, development, physiology and defense (Pieterse *et al*., 2012; J. Y. Li *et al*., 2017). While different phytohormones have distinct biological functions, they may also function synergistically, additively or antagonistically (Mur *et al*., 2006; Pieterse *et al*., 2012; J. Y. Li *et al*., 2017). Phytohormones are central cellular signal molecules with key functions in the regulation of plant immunity against biotic stimuli, including viruses, microbial pathogens and insect herbivores (Pieterse *et al*., 2012). While many phytohormone pathways including abscisic acid (ABA) have been implicated in regulating plant immunity against biotic stimuli, most studies have focused on the jasmonate (JA), salicylic acid (SA) and ethylene (ET) pathways (Wu & Baldwin, 2010; Pieterse *et al*., 2012; Alazem & Lin, 2015; Broekgaarden *et al*., 2015; Verma *et al*., 2016; Zhang *et al*., 2017).

In this article, we first review studies of the effects of virus infection on plant–insect interactions as modulated by the three intensively investigated phytohormones: JA, SA and ET. We then examine the effects of virus infection on phytohormones other than JA, SA and ET. Next, we address the role of factors that may affect virus‐induced modification of these phytohormone pathways and plant–insect interactions. We then elaborate on issues of particular relevance to investigation of the changes to phytohormone pathways induced by plant viruses and their effects on plant–insect interactions. Finally, we speculate on future directions of research to disentangle the complex tripartite interactions and factors involved.

## II. Phytohormone‐mediated effects of virus infection on plant–insect interactions

Jasmonate, SA and ET condition plant‐defense responses against abiotic and biotic stresses (Pieterse *et al*., 2012; Verma *et al*., 2016). In general, the JA‐signaling pathway confers a broad‐spectrum resistance to necrotrophic pathogens and insect herbivores. The SA‐signaling pathway plays a major role in disease resistance signaling by countering the invasions of biotrophic microbial pathogens, including viruses. The JA and SA pathways are often antagonistic to each other, as activation of JA signaling frequently leads to suppression of SA signaling and vice versa (Pieterse *et al*., 2012; Zhang *et al*., 2017). Unlike JA and SA, both of which are usually directly involved in mediating plant responses, ET often functions indirectly by regulating other plant hormone pathways (Leon‐Reyes *et al*., 2009; Pieterse *et al*., 2012; Broekgaarden *et al*., 2015). However, in some cases, components of the ET‐signaling pathway may directly regulate plant defense against biotic attackers (McGrath *et al*., 2005; Liu *et al*., 2011; Lu *et al*., 2011). For example, manipulation of ET‐response transcription factors in Arabidopsis results in altered resistance to several fungi; similarly, manipulation of the expression of an ET synthase gene results in altered plant resistance to *Chilo suppressalis* (McGrath *et al*., 2005; Lu *et al*., 2011). The flux of these major defense‐related signaling pathways and the molecular mechanisms underlying their interactions have been comprehensively reviewed by Pieterse *et al*. (2012), Broekgaarden *et al*. (2015) and Zhang *et al*. (2017). Here we concentrate on the effects of virus infection on plant–insect interactions via modulation of the JA‐, SA‐ and ET‐signaling pathways (Table 1).

**Table 1:** Phytohormone‐mediated effects of virus infection on plant–insect interactions.

Pathosystem (virus–plant–insect)	Insect performance/preference	Phytohormone biosynthesis/catabolism and signaling	Viral effector	Mechanism	References
Tomato yellow leaf curl China virus–*Nicotiana tabacum*–*Bemisia tabaci*	Increased insect performance	Downregulated JA biosynthesis/catabolism and signaling	βC1	Interaction between βC1 and MYC2	Jiu *et al*. (2007), Zhang *et al*. (2012), Luan *et al*. (2013), Li *et al*. (2014)
Tomato yellow leaf curl virus–*Solanum lycopersicum*‐*B. tabaci*	Increased insect performance	Downregulated JA signaling	?	Disruption of JA downstream defenses	Su *et al*. (2016)
Cucumber mosaic virus–*Arabidopsis thaliana*–*Myzus persicae*	Increased insect preference	Downregulated JA signaling	2b	Interaction between 2b and JAZ	Wu *et al*. (2017)
Tomato yellow leaf curl virus‐*N. tabacum*–*B. tabaci*	Increased insect performance	Downregulated JA signaling	C2	Interaction between C2 and ubiquitin	Li *et al*. (2019)
Tomato spotted wilt orthotospovirus–*Capsicum annuum*–*Frankliniella occidentalis*	Increased insect performance and preference	Downregulated JA signaling	NSs	Interaction between NSs and MYC2	Wu *et al*. (2019)
Cotton leaf curl Multan virus and tomato yellow leaf curl China virus–*Arabidopsis thaliana*–*B. tabaci*	Increased insect performance	Downregulated JA signaling	βC1	Interaction between βC1 and WRKY20	Zhao *et al*. (2019)
Potato leafroll virus–*Solanum tuberosum* and *Nicotiana benthamiana*–*M. persicae*	Increased insect performance and preference	Downregulated JA biosynthesis/catabolism	P0, P1 and P7	?	Patton *et al*. (2019)
Tobacco mosaic virus–*Solanum lycopersicum*–*Spodoptera exigua*	Increased insect performance	Upregulated SA biosynthesis/catabolism	?	?	Thaler *et al*. (2010)
Tomato spotted wilt virus–*S. lycopersicum*–*Tetranychus urticae*	Increased performance and preference	Upregulated SA biosynthesis/catabolism and signaling	?	?	Nachappa *et al*. (2013)
Potato virus Y–*Solanum lycopersicum*–*Macrosiphum euphorbiae and Leptinotarsa decemlineata*	Increased insect performance	Upregulated SA biosynthesis/catabolism	?	?	Kersch‐Becker & Thaler (2014)
Tomato spotted wilt virus–*Arabidopsis thaliana*–*Frankliniella occidentali*	Increased insect preference	Upregulated SA signaling	?	?	Tomitaka *et al*. (2015)
Pea enation mosaic virus–*Pisum sativum*–*Sitona lineatus*	Increased insect preference	Upregulated SA biosynthesis/catabolism	?	?	Chisholm *et al*. (2018)
Tomato mosaic virus–*S. lycopersicum*–*B. tabaci*	Decreased insect preference	Upregulated SA biosynthesis/catabolism and signaling	?	?	Ueda *et al*. (2019)
Tomato spotted wilt virus–*A. thaliana*–*Frankliniella occidentalis*	Increased insect performance and preference	Upregulated SA biosynthesis/catabolism and signaling and downregulated JA signaling	?	?	Abe *et al*. (2008, 2012)
Turnip mosaic virus–*Arabidopsis thaliana*–*M. persicae*	Increased insect performance	Upregulated ET biosynthesis/catabolism	NIa‐Pro	?	Casteel *et al*. (2014, 2015)
Potato virus Y–*Solanum tuberosum*–*M. persicae*	Increased insect preference	Upregulated ET biosynthesis/catabolism	?	?	Bak *et al*. (2019)
Potato leafroll virus–*Solanum tuberosum* and *Nicotiana benthamiana*–*M. persicae*	Increased insect performance and preference	Downregulated ET biosynthesis/catabolism	P0, P1 and P7	?	Patton *et al*. (2019)

### 1. Jasmonates

**Fig. 1:**
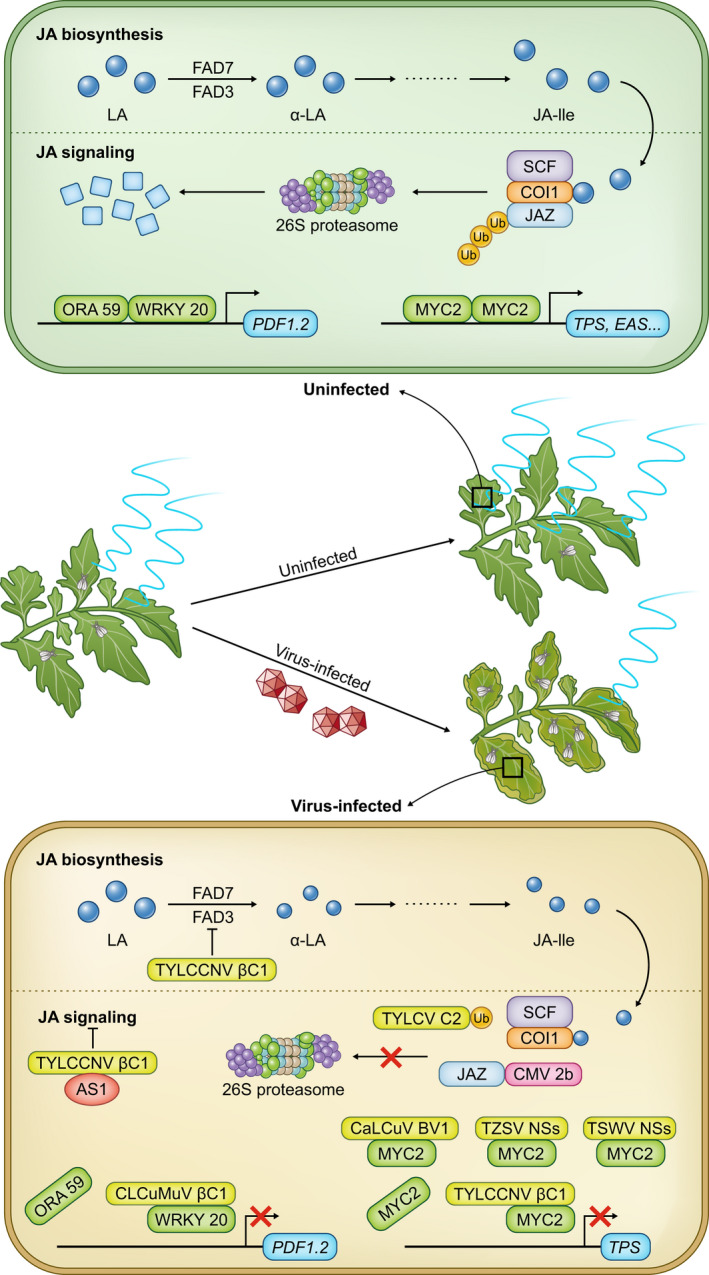
Schematic representation of the jasmonate (JA)‐mediated manipulation of plant–insect interaction by viruses. In uninfected plants, infestation of plants by insects leads to activation of JA biosynthesis/catabolism and signaling, which in turn reduce the population of insects. Schematic overview of the flux of JA biosynthesis/catabolism and signaling is modified from Browse (2009) and Pieterse *et al*. (2012). Biosynthesis of JA starts with linolenic acid (LA), which is converted to α‐LA by FAD3 and FAD7, and finally to JA‐isoleucine (JA‐Ile). Upon JA‐Ile synthesis, JAZ proteins are ubiquitinated and degraded by 26S proteasomes, thereby relieving JAZ‐mediated suppression of MYC2. MYC2 then forms an active dimer to activate the expression of downstream genes such as *terpene synthase* (*TPS*) and *epi‐arisotolchene synthase* (*EAS*) genes, and in turn the synthesis of volatiles, including terpenoids. In addition, activation of JA‐signaling pathway results in interaction between ORA59 and WRKY20, which in turn activates the expression of downstream genes, such as *PLANT DEFENSIN 1.2* (*PDF1.2*). However, in virus‐infected plants, virus infection compromises the activation of JA biosynthesis/catabolism and signaling at several levels, resulting in a reduction in the biosynthesis and release of volatiles and an increase in the insect population. Specifically, βC1 encoded by tomato yellow leaf curl China virus (TYLCCNV) downregulates the expression of *FAD3* and *FAD7* and interacts with MYC2 and AS1 to interfere with JA biosynthesis/catabolism and signaling, respectively (Yang *et al*., 2008; Luan *et al*., 2013b; Li *et al*., 2014). C2 encoded by tomato yellow leaf curl virus (TYLCV) and 2b encoded by cucumber mosaic virus (CMV) interact with ubiquitin and JAZ, respectively, thereby preventing the degradation of JAZ to interfere with JA signaling (Wu *et al*., 2017; Li *et al*., 2019). In addition, BV1 encoded by cabbage leaf curl virus (CaLCuV), NSs encoded by tomato spotted wilt virus (TSWV), and tomato zonate spot virus (TZSV) bind to MYC2, thereby preventing the dimerization of MYC2 and activation of downstream gene expression (Li *et al*., 2014; Wu *et al*., 2019). βC1 encoded by cotton leaf curl Multan virus (CLCuMuV) binds to WRKY20 to interfere with the interaction between WRKY20 and ORA59 and, in turn, the expression of downstream genes (Zhao *et al*., 2019).

Jasmonate is commonly considered in studies of the effects of virus infection on plant–insect interactions, mainly as a result of its direct involvement in plant defense against insect herbivores and production of a volatile blend (Mauck *et al*., 2010; Zhang *et al*., 2012; Li *et al*., 2014; Su *et al*., 2016; Wu *et al*., 2017; Wu & Ye, 2020). Many components of the JA‐signaling pathway are manipulated by viruses, thereby impacting plant–insect interactions (Fig. 1). Infection by begomoviruses suppresses JA biosynthesis/catabolism (resulting in lower levels of accumulation of JA) and/or signaling, leading to enhanced performance of their whitefly vectors (Zhang *et al*., 2012; Luan *et al*., 2013b; Su *et al*., 2016; P. Li *et al*., 2019). Infection of tobacco by tomato yellow leaf curl China virus (TYLCCNV) promotes the performance of its whitefly vector (Jiu *et al*., 2007). This enhanced whitefly performance is a result of the viral satellite βC1 protein, which suppresses JA biosynthesis/catabolism, JA signaling and terpenoid biosynthesis/catabolism (Zhang *et al*., 2012; Luan *et al*., 2013b). Mechanistically, interactions between βC1 and several plant proteins are known, including three transcription factors (MYC2, WRKY20 and PHYTOCHROME‐INTERACTING FACTOR) and the S‐phase kinase‐associated protein 1 (Li *et al*., 2014; Zou *et al*., 2020; Zhao *et al*., 2019, 2021). Similarly, the infection by tomato yellow leaf curl virus (TYLCV) in tomato interferes with JA signaling, leading to suppression of plant defenses and, in turn, enhanced performance of its whitefly vector (Su *et al*., 2016). A more recent study with TYLCV and tobacco shows that the viral C2 protein inhibits JA signaling using a distinct mechanism. C2 binds to the N‐terminal ubiquitin domain of the tobacco 40S ribosomal protein RPS27A *in vivo* and *in vitro*. The C2–RPS27A interaction compromises the degradation of JAZ1, a negative regulator of JA signaling, resulting in inhibition of JA signaling and enhanced performance of the whitefly vector (P. Li *et al*., 2019).

Manipulation of the JA‐signaling pathway by viruses may affect the attractiveness of plants. For example, squash plants infected by cucumber mosaic virus (CMV) become more attractive to its aphid vectors, *Myzus persicae* and *Aphis gossypii* (Mauck *et al*., 2010). The 2b protein of CMV, which is associated with the increased preference of aphid vectors, disrupts the JA‐signaling pathway in plants (Lewsey *et al*., 2010; Ziebell *et al*., 2011). Profiling of volatiles revealed that 2b protein induces quantitative and qualitative changes in the insect‐perceivable blends of volatiles emitted by plants (Groen *et al*., 2016). The viral 2b protein interacts directly with JAZ proteins to prevent JA‐induced JAZ degradation, thereby attenuating JA signaling and increasing the allure of CMV‐infected plants to aphids (Groen *et al*., 2016; Wu *et al*., 2017).

Several studies have used genetic approaches to elucidate the effects of endogenous JA on viral modulation of plant–insect interactions. Using wild‐type Castlemart, JA‐deficient *spr2* mutant and JA‐overexpression *35S‐prosystemin* transgenic tomato plants, Sun *et al*. (2017) observed that whiteflies feeding on TYLCV‐infected plants exhibit enhanced survival and reproduction on 35S‐*prosystemin* plants as compared with uninfected plants. Similarly, Liu *et al*. (2017) demonstrated that TYLCV‐infected whiteflies feed more extensively on wild‐type and JA‐deficient plants relative to uninfected whiteflies, but feeding differences are not evidenced on the *35S‐prosystemin* plants.

### 2. Salicylic acid

The SA pathway is often affected by virus infection, resulting in altered plant–insect interactions. Shi *et al*. (2016) found that with the increase of CMV titer, SA biosynthesis/catabolism and signaling are induced and aphid performance is correspondingly depressed. Thaler *et al*. (2010) found that infection of tomato by tobacco mosaic virus induces SA biosynthesis/catabolism (resulting higher levels of accumulation of JA) but suppresses JA biosynthesis/catabolism, resulting in increases of the growth of *Spodoptera exigua* caterpillars in a SA‐dependent manner. Kersch‐Becker & Thaler (2014) reported that potato virus Y (PVY) infection of tomato plants induces SA biosynthesis/catabolism, which is correlated with enhanced performance of an aphid vector and two nonvector insects of PVY. Finally, a mechanism for virus‐induced SA increases was provided by Zhao *et al*. (2019). They showed that the expression of βC1 encoded by cotton leaf curl Multan virus (CLCuMuV) activates SA biosynthesis/catabolism and signaling by targeting WRKY20; this interaction renders Arabidopsis plants more resistant to the aphid *M. persicae*.

The SA‐signaling pathway may also influence virus‐induced host attractiveness to insect herbivores. Tomitaka *et al*. (2015) showed that tomato spotted wilt virus infection renders plants more attractive to its insect vector *Frankliniella occidentalis*; the increased attractiveness is associated with upregulated SA signaling. Similarly, Chisholm *et al*. (2018) showed that pea enation mosaic virus infection of pea plants makes the plants more attractive to *Sitona lineatus*, and this elevated attractiveness is correlated with virus‐induced increases in SA.

### 3. JA–SA antagonism

The antagonistic relationship of the JA‐ and SA‐signaling pathways may modulate the interactions between insects and virus‐infected plants. Preston *et al*. (1999) reported that infection by tobacco mosaic virus induces a systemic increase in SA and attenuates wound‐induced JA and nicotine responses of *Nicotiana attenuata* to promote leaf consumption by *Manduca sexta* larvae. In tomato plants, begomovirus infection also causes increases in SA biosynthesis/catabolism and signaling, and downregulation of JA biosynthesis/catabolism and signaling (Cui *et al*., 2016). Similarly, Abe *et al*. (2008, 2012) showed that infection by tomato spotted wilt virus results in elevation of SA biosynthesis/catabolism and signaling with simultaneous suppression of JA signaling, leading to increases in thrips preference for and performance on virus‐infected plants. These data, as well as the studies highlighted in the JA and SA sections, stress the importance of phytohormone dynamics for the relative success of the three partners in each tripartite interaction.

### 4. Ethylene

A small number of studies show that the ET‐signaling pathway is modulated during viral infection and impacts plant–insect interactions. Casteel *et al*. (2014, 2015) showed that infection of Arabidopsis plants by turnip mosaic virus enhances performance of its aphid vector *M. persicae*. The improved vector performance is attributed to the disruption of ET signaling by the virus‐encoded Nuclear Inclusion a‐Protease (Nla‐Pro), which reduces callose deposition and leads to increased plant palatability for the aphid vector. By contrast, Bak *et al*. (2019) reported that the preference of *M. persicae* to settle on PVY‐ and turnip mosaic virus‐infected potato plants over uninfected ones is associated with the induction of ET biosynthesis/catabolism by the virus. These findings suggest that the role of ethylene in the manipulation of plant–insect interactions by viruses may be host plant‐specific and this provides ample opportunities for future investigation.

## III. Effects of virus infection on phytohormones other than JA, SA and ET

In addition to the primary plant defense hormones JA, SA and ET, other plant hormones, such as auxin, gibberellins (GAs), cytokinins (CKs), brassinosteroids (BRs), and abscisic acid (ABA), may be affected by viral infection. However, the impacts of these plant hormones on viral–plant–insect interactions have received limited attention (Fraser & Whenham, 1982; Jameson & Clarke, 2002; Benjamins & Scheres, 2008; Alazem & Lin, 2017). Of significance are the notable symptoms caused by virus infection that resemble the phenotypes of biosynthesis or signaling mutants of the auxin, GA, CK, BR and ABA pathways (Jameson & Clarke, 2002; Kazan & Manners, 2009; Satoh *et al*., 2011; Mach, 2012; Yifhar *et al*., 2012).

### 1. Auxin

Auxin is a key factor in regulating plant growth and development (Benjamins & Scheres, 2008). Viral infections of plants lead to severe developmental abnormalities, such as stunting, leaf curling and loss of apical dominance, suggesting that host auxin homeostasis/signaling could be modulated by viral infection. As early as the 1930s, the relationship between auxins and virus infection was investigated. The role of auxins in response to virus infection is complex, as both declines and increases in the expression of auxin pathway genes are documented (Fraser & Whenham, 1982; Jameson & Clarke, 2002). Leng *et al*. (2017) reported that sugarcane mosaic virus infection increases the expression of the *Auxin binding protein 1* (*ABP1*) gene. While the role of ABP1 in auxin signaling has yet to be resolved (Papanov *et al*., 2019), ABP1 is associated with viral resistance in maize. In addition, an increasing number of studies have proposed the manipulation of auxin response factors (ARFs) by viruses; ARFs are a group of transcription factors that translate auxin signals to downstream gene expression (Guilfoyle & Hagen, 2007) and they account for the phenotypic abnormalities caused by viral infection. The expression of tomato *ARF4*, *ARF5*, *ARF6A*, *ARF8B* and *ARF9A* genes is downregulated in leaves infected by TYLCV; sequence analyses indicate that 5′‐regulatory regions of these *ARF*s are enriched in biotic and abiotic stress‐responsive *cis*‐elements (Bouzroud *et al*., 2018). Similarly, in response to rice dwarf virus infection, rice plants suppress genes involved in the early steps of indole‐3‐acetic acid synthesis (e.g. *ARF*s and many auxin‐responding *SMALL AUXIN UP RNA* genes) (Satoh *et al*., 2011).

### 2. Gibberellins

Gibberellins, another class of crucial hormones that function in the regulation of plant growth, are modulated by virus infection (Zhu *et al*., 2005; Robert‐Seilaniantz *et al*., 2007; Satoh *et al*., 2011; Wang *et al*., 2011; Tao *et al*., 2017). The infection of nonheading Chinese cabbage by turnip mosaic virus results in a reduction in endogenous GA concentrations and expression of GA‐regulated genes (Wang *et al*., 2011). Similarly, rice dwarf virus infection suppresses the expression of GA biosynthesis genes and induces expression of genes involved in GA inactivation (Zhu *et al*., 2005; Satoh *et al*., 2011). Mechanistic insights were provided by Zhu *et al*. (2005). During rice dwarf virus infection in rice plants, the viral outer capsid protein P2 interacts with *ent*‐kaurene oxidases, which are crucial to the biosynthesis of GAs; this results in an inhibition of *ent*‐kaurene oxidase activity and a subsequent decline of GA concentrations, leading to stunting and other associated symptoms (Zhu *et al*., 2005). Furthermore, exogenous application of GA_3_ to rice dwarf virus‐infected plants rescues the abnormal growth phenotypes, an observation consistent with that of Maramorosch (1957). Finally, rice black‐streaked dwarf virus regulates the GA‐signaling pathway via the interaction of the virus‐encoded P7‐2 and the plant’s GA‐insensitive dwarf2 (GID2) protein, which is an important component of GA signaling that dictates the degradation of DELLA proteins (Tao *et al*., 2017). DELLAs are critical regulatory proteins that help to mediate crosstalk of GA with the JA‐ and SA‐signaling pathways, which is essential for balancing growth and defense (Navarro *et al*., 2008; Y. Li *et al*., 2019).

### 3. Cytokinins

In response to virus infection, changes in plant CK concentrations may occur. While infection of *Phaseolus vulgaris* plants by white clover mosaic potexvirus results in a reduction in active CKs (Clarke *et al*., 1999), CMV infection of *Arabidopsis thaliana* seedlings leads to increased and unaltered CK metabolism in roots and shoots, respectively (Vitti *et al*., 2013). Baliji *et al*. (2010) found that geminivirus pathogenicity proteins interact with and inhibit an adenosine kinase, which maintains a pool of bioactive CKs; this leads to increased expression of primary CK‐responsive genes and reprogramming of the plant cell cycle to enable geminivirus replication. This finding is further confirmed by the observation that treatment with a CK increases the susceptibility of plants to geminivirus infection (Baliji *et al*., 2010). In view of the disparate CK responses to viruses and an emerging, but limited, understanding of CKs in plant defense (Akhtar *et al*., 2020), research into the role of CKs in tripartite interactions is timely.

### 4. Brassinosteroids

Brassinosteroids play vital roles in regulating plant growth and stress responses (Divi & Krishna, 2009; Nolan *et al*., 2017). However, little is known about the response of BRs to virus infection. Recently some advances have been made. Bi *et al*. (2017) reported that the C4 protein of sweet potato leaf curl virus interacts with the *A. thaliana* brassinosteroid‐insensitive 2 (AtBIN2) protein. This interaction leads to nuclear translocation of two AtBIN2‐interacting proteins (AtBES1 and AtBZR1), which are transcription factors controlling the expression of most BR‐responsive genes. The role of BRs in tripartite interactions is a virtually unexplored territory and should be prioritized, given that BZR1 and BAK1 (Brassinosteroid insensitive1‐Associated Receptor Kinase 1) have roles in defense against insects and have links to JA signaling, as well as balancing defense and growth in plants (Miyaji *et al*., 2014; Prince *et al*., 2014; Yu *et al*., 2018).

### 5. Abscisic acid

The role of ABA in regulating abiotic stress tolerance is well known, and ABA also plays a crucial role in the response of plants to pathogen infection by regulating stomata opening/closing (Ton *et al*., 2009). However, case studies on the modulation of ABA by viruses have yielded inconsistent results. Whenham *et al*. (1986) observed increases in ABA in leaves of tobacco (*N. tabacum*) systemically infected by tobacco mosaic virus. Similarly, Alazem *et al*. (2014) found that infection of *N. benthamiana* by bamboo mosaic virus or CMV and Arabidopsis by bamboo mosaic virus increases ABA content and the mRNAs of several genes in the ABA pathway. By contrast, the ABA pathway is downregulated during rice black‐streaked dwarf virus infection of rice and CMV infection of Arabidopsis (Westwood *et al*., 2013b; Xie *et al*., 2018).

## IV. Additional factors involved in virus‐induced changes of the tripartite interactions

A limited number of experimental studies have been conducted on the additional factors that influence the virus‐induced changes of plant hormone pathways associated with plant–insect interactions. Major factors examined in these studies include plant nutrition, plant secondary metabolites and natural enemies of insects.

### 1. Nutrition

The nutritional quality of host plants is a key factor determining the growth and reproduction of phytophagous insects (Awmack & Leather, 2002). Infection by plant viruses may have positive, neutral or negative effects on the quantity/profile of nutrients in plants and, in turn, the performance of insects. For example, Su *et al*. (2015) showed that TYLCV infection of tomato plants results in higher concentrations of sugars and amino acids in the phloem sap, which may account for the increased performance of a whitefly vector of the virus. Similarly, infection of squash plants by papaya ringspot virus increases the concentrations of several free essential and nonessential amino acids correlating with enhanced performance of its aphid vector *A. gossypii* (Gadhave *et al*. 2019). By contrast, He *et al*. (2014) showed that the infection of rice plants by southern rice black‐streaked dwarf virus does not significantly change amino acid or soluble sugar content. Finally, at the other end of the spectrum, Fiebig *et al*. (2004) reported a significant reduction in total amount of amino acids in wheat plants infected by barley yellow dwarf virus, which may result in the reduced nutritional assimilation by the aphid *Sitobion avenae* and its poor overall performance.

In some cases, virus infection may not affect the quantity or profile of plant nutrients. Instead the uptake of nutritional compounds by insects may be altered. Wang *et al*. (2012) showed that virus infection of tobacco does not change the nutrient profile in the phloem sap. However, whiteflies feeding on tobacco plants infected by TYLCCNV excrete a lower percentage of amino acids, especially essential amino acids, and, proportionally, a higher concentration of sugars than whiteflies feeding on uninfected plants. These data suggest that whiteflies assimilate more amino acids from TYLCCNV‐infected plants. Plant viruses may also alter the nutritional status of certain organs/tissues of plants to manipulate the behavior of insect vectors to maximize virus dissemination. For example, CMV infection decreases the ratio of simple carbohydrates to amino acids in mesophyll and epidermal cells, the sites that aphids initially probe and acquire virions, thus promoting vector feeding and virus acquisition (Mauck *et al*., 2010, 2014). Reciprocally, in the phloem, where aphids establish long‐term feeding sites, CMV infection results in decreased nutritional quality to promote the dispersal of viruliferous aphids (Mauck *et al*., 2010, 2014).

### 2. Volatile organic compounds and other secondary metabolites

Plants synthesize a broad range of secondary metabolites, many of which are released as volatiles to impact herbivores and their natural enemies. Volatiles emitted by virus‐infected plants play a major role in determining the settling and feeding preference of insect vectors (de Vos & Jander, 2010; Mauck *et al*., 2012, 2018). For example, winged adults of *M. persicae* preferentially settle on potato plants infected by potato leafroll virus (Castle *et al*., 1998). This preference is associated with the different volatile profiles emitted by virus‐infected and uninfected plants (Eigenbrode *et al*., 2002). In addition, a specific volatile blend is responsible for arresting *M. persicae* on potato leafroll virus‐infected plants (Ngumbi *et al*., 2007). CMV‐infected squash plants emit larger quantities of volatiles, although no major qualitative changes of the volatile blend occur; these volatiles attract the insect vectors of CMV, *M*. *persicae* and *A. gossypii* (Mauck *et al*., 2010). However, the impact of changes on the volatile blends varies with different host plants. For instance, CMV infection of common tobacco induces both quantitative and qualitative changes in plant volatile emissions but does not alter the settling preference of the aphid *M*. *persicae* (Tungadi *et al*., 2017).

While some studies show that the blend of volatiles determines insect preference to plants, virus‐induced attraction may be attributed to a few or even a single volatile organic compound. Nonviruliferous wingless adults of the aphid *Rhopalosiphum padi* preferentially settle on wheat plants infected by barley yellow dwarf virus as compared with uninfected plants (Jiménez‐Martínez *et al*., 2004). Wheat plants infected by barley yellow dwarf virus emit larger quantities of several volatiles, including nonanal, (Z)‐3‐hexenylacetate, decanal, β‐caryophyllene and undecane. These volatiles are associated with the increased preference of nonviruliferous, wingless aphid adults to virus‐infected plants (Medina‐Ortega *et al*., 2009). Infection of white clover plants by clover mosaic virus increases the quantity of several volatiles in the volatile blend emitted by plants (such as β‐caryophyllene), and this increase is associated with a decreased attractiveness to adult fungus gnats (van Molken *et al*., 2012). The infection of red raspberry *Rubus idaeus* by black raspberry necrosis virus and raspberry leaf mottle virus increases the quantity of (Z)‐3‐hexenyl acetate in the plant volatile blend. This leads to the preference of the large raspberry aphid, *Amphorophora idaei*, for virus‐infected plants (McMenemy *et al*., 2012).

Apart from insect preference for plants, secondary metabolites are implicated in virus modulation of other aspects of plant–insect interactions. Martins *et al*. (2012) showed a positive association between the severity of sticky disease symptoms caused by papaya meleira virus and the infestation frequency of Mediterranean fruit fly. Virus‐infected papaya plants contain reduced concentrations of benzylisothiocyanate, a compound with putative resistance properties to fruit fly, presumably making virus‐infected plants more palatable to fruit fly. Similarly, Westwood *et al*. (2013a) showed that infection of Arabidopsis plants by CMV induces the biosynthesis/catabolism of the aphid‐feeding deterrent 4‐methoxy‐indol‐3‐yl‐methylglucosinolate (4MI3M), thereby inhibiting phloem ingestion by the aphid *M. persicae*.

In some cases, virus infection may attenuate or compromise the ability of a host plant to synthesize secondary metabolites that contribute to plant resistance to insects. For example, Luan *et al*. (2013b) and Li *et al*. (2019, 2014) showed that whitefly infestation induces terpenoid biosynthesis/catabolism in plants; however, terpenoid biosynthesis/catabolism is decreased in begomovirus‐infected plants, leading to an increased palatability of host plants to whiteflies. Similarly, soybean mosaic virus infection of soybean plants suppresses aphid‐induced terpene biosynthesis/catabolism (Laney *et al*., 2018). Furthermore, plant viruses may differentially modulate the biosynthesis of natural compounds in different plant tissues. Zhao *et al*. (2019) showed that through its interactions with the transcription factor WRKY20, the βC1 of CLCuMuV differentially regulates glucosinolate biosynthesis/catabolism in nonvein leaf tissues and leaf veins of Arabidopsis plants. The absence of JA‐regulated defenses leads to enhanced performance of the whitefly on CLCuMuV‐infected plants relative to uninfected plants.

### 3. Natural enemies of insect vectors

Infestation of plants by insect herbivores may affect the activities of natural enemies, which function as an indirect defense against insect herbivores (de Vos & Jander, 2010). These indirect defenses can be modified by virus infection of the host plant. Working with tomato, TYLCV, whiteflies and the parasitoid *Encarsia formosa*, Liu *et al*. (2018) showed that the parasitoid prefers TYLCV‐infected plants to uninfected plants, resulting in a higher parasitism rate of whiteflies on TYLCV‐infected plants. A very different outcome was noted by Belliure *et al*. (2008) when studying tomato spotted wilt virus. In this case, viral infection increases the growth rate of *F. occidentalis*, resulting in a reduced period of vulnerability of the thrips to predation by two species of predatory mites, as large larvae are not susceptible to mite predation. By contrast, several studies show that viruses do not alter natural enemy visitations or their ability to attack prey. For example, Joffrey *et al*. (2018) showed that the infection of *Camelina sativa* plants by turnip yellows virus does not render the plants more attractive or repellent to an aphid parasitoid *Aphidius colemani*. Similarly, CMV infection of squash has no significant impact on the ability of predatory insects to locate aphid prey (Mauck *et al*., 2015). Finally, infection of rice by southern rice black‐streaked dwarf virus does not change the capability of *Anagrus nilaparvatae* to parasitize eggs of the brown planthopper *Nilaparvata lugens* (He *et al*., 2014).

## V. Perspectives

The case studies available to date indicate that the effects of viruses on phytohormones and plant–insect interactions vary with many genetic and environmental variables (Mauck *et al*., 2012; Eigenbrode *et al*., 2018). We are at the beginning of unraveling the underlying behavioral, physiological and molecular mechanisms that dictate these interactions (Wang & Blanc, 2021). Any breakthrough in this area of study will require a multidisciplinary and integrated approach. We discuss below some of the issues that call for particular attention in future investigations.

### 1. Complexity and variability of the tripartite interactions

Complexity and variability of the tripartite interactions between viruses, plants and insects are dictated by genetic and environmental variables. Intrinsically, these interactions are shaped by the genotypes and traits of each of the three players. The replacement of even one of the three players with another member of the same group may result in dramatic changes in the tripartite interaction. For example, PVY infection in potato plants promotes feeding of the aphid *M. persicae*, but reduces feeding of the aphid *Macrosiphum euphorbiae* (Boquel *et al*., 2011). While CMV‐infected Arabidopsis plants are more attractive to *M. persicae*, the virus does not have marked effects on the preference of *M. persicae* when tobacco plants are used (Tungadi *et al*., 2017; Wu *et al*., 2017).

Even replacement of one strain/line with another of the same species may incur substantial changes in the tripartite interaction. Using three tomato lines that vary in the strength of the JA‐signaling pathway, Sun *et al*. (2017) found that virus infection of plants enhanced whitefly performance thereon more frequently and at a higher level when plants with relatively high JA‐regulated defenses were used. Considering the large number of alternative species/strains of each player (plant, insect and virus), the number of potentially distinct tripartite interaction combinations is vast. For example, over 400 species of begomoviruses (family *Geminiviridae*; genus *Begomovirus*) are known and, in theory, each of them can be transmitted by one or several species of whiteflies of the *Bemisia tabaci* complex, which consists of > 40 whitefly species (De Barro *et al*., 2011; Liu *et al*., 2012; Zerbini *et al*., 2017; Kanakala & Ghanim, 2019; Wang & Blanc, 2021; https://talk.ictvonline.org/ictv‐reports/ictv_online_report/ssdna‐viruses/w/geminiviridae, accessed 26 October 2020). In addition, many of these viruses and their whitefly vectors have a wide range of host plants (Mansour & Al‐Muse, 1992; Malka *et al*., 2018). Thus, one can only imagine how many tripartite combinations of begomoviruses, plants and whiteflies may arise.

Environmentally, other variables, either biotic (i.e. coexisting herbivores, pathogens and natural enemies) or abiotic (i.e. temperature, moisture and light), may modify the dynamics and outcomes of tripartite interactions. These variables may alter virus–plant–insect interactions directly by affecting the interaction between any two of the three players or indirectly by altering the performance of one of the organisms. This premise is supported by the fact that even alternating the sequence of biotic challenges encountered by a host plant substantially modifies the tripartite interaction. For example, a change from ‘virus first and then insect vector’ to ‘insect vector first and then virus’ in laboratory experiments, alters the tripartite interaction, both qualitatively and quantitatively (P. Li *et al*., 2017). Furthermore, in natural or cultivated ecosystems, biological invasion or change of agricultural practices may lead to changes to the combinations of the three partner organisms and, subsequently, their associated natural enemies. This has the potential to create unprecedented food webs, adding another degree of complexity and variability to tripartite interactions.

When discussing the modulation of plant–insect interactions by viruses, attention has mostly focused on the biological characteristics of viruses, such as the mechanisms (persistent vs nonpersistent) underlying virus transmission by insect vectors (Mauck *et al*., 2012, 2018; Eigenbrode *et al*., 2018). However, as discussed earlier, many genetic and environmental variables may contribute to the modulation of plant–insect interactions by viruses. Thus, interpretation of experimental studies and natural observations on the tripartite interactions should always take these factors into consideration. Many more case studies using pathosystems, either naturally occurring or artificial, in both laboratory and field scales are required to elucidate the general principles that govern tripartite interactions.

### 2. Strategies to reveal the underlying mechanisms of tripartite interactions

**Fig. 2:**
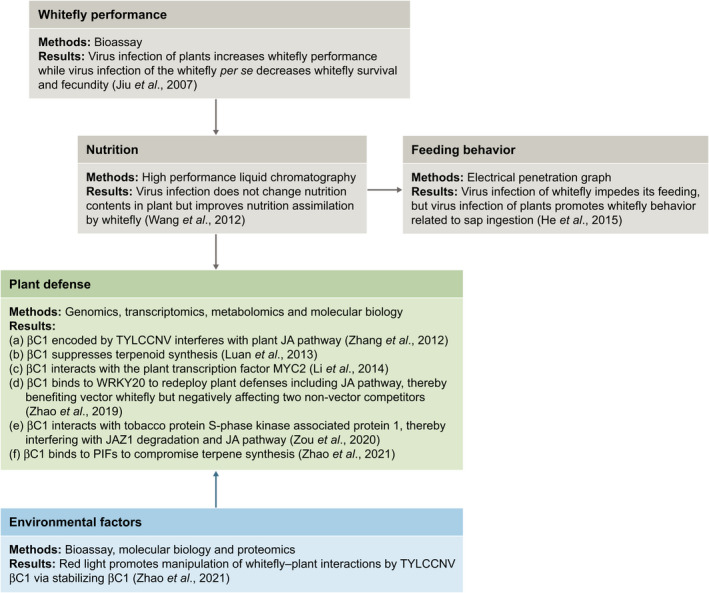
Flowchart of dissection of the mechanisms underlying the tripartite interactions among tomato yellow leaf curl China virus (TYLCCNV), MEAM1 whitefly and tobacco plants. Initially, research focused on plant nutritional content. These data led to the investigation of whitefly feeding behavior and, more importantly, plant defenses. The physiological and molecular mechanisms underlying the virus‐induced changes in phytohormones and plant defense/immunity are investigated by integrating genetics, genomics, transcriptomics and metabolomics using cutting‐edge technologies. This research was then extended to examine the effects of environmental factors, such as light on the tripartite interactions.

While the mechanisms underlying tripartite interactions involving plant, virus and insect vector are complex and variable, they can be tractable when an appropriate strategy is adopted for their study. To date, a relatively small number of tripartite systems have been intensively investigated. Therefore, we present an example of the extensive and collaborative work that has focused on unraveling the intricacies in tripartite interactions involving begomoviruses. In the past decade, a joint effort from our laboratory and the laboratories of Professor Xue‐Ping Zhou (Zhejiang University) and Professor Rong‐Xiang Fang (Institute of Microbiology, Chinese Academy of Sciences) has begun to reveal the complexities of the interactions among begomoviruses, whitefly vectors and their shared host plants (Jiu *et al*., 2007; Zhang *et al*., 2012; Wang *et al*., 2012, 2020; Luan *et al*., 2013a,b; Li *et al*., 2014; He *et al*., 2015; P. Li *et al*., 2017, 2019; Zhao *et al*., 2019, 2021; He *et al*., 2020; Wang *et al*., 2020; Zou *et al*., 2020). Our complementary studies demonstrate the feasibility of unraveling the underlying mechanisms, in particular the modulation and function of phytohormones, using a multidisciplinary, integrated approach that utilizes cutting‐edge technologies (Fig. 2). Collectively, these efforts vividly demonstrate the complexity of the interaction network in a tripartite system.

In this joint effort, the tripartite system consisting of tobacco plants, TYLCCNV and two species of whiteflies of the *B. tabaci* complex (MEAM1 and MED) has received most attention. Initial bioassays showed that whitefly performance and population increases are enhanced on virus‐infected plants relative to uninfected plants. However, this positive effect on whiteflies is not a result of virus infection of the whiteflies *per se* but rather of the feeding of whiteflies on virus‐infected plants (Jiu *et al*., 2007). In fact, virus infection of whiteflies reduces survival and fecundity (Jiu *et al*., 2007). LC profiling of the amino acids and sugar content of plants, whiteflies and whitefly honeydew shows that virus infection of host plants results in little change of the nutritional content of the phloem sap. However, compared with whiteflies feeding on uninfected plants, whiteflies on virus‐infected plants excrete a significantly higher proportion of sugar and a lower proportion of amino acids, particularly some essential amino acids. The lower amino acid content of the honeydew suggests that whiteflies have enhanced nutrient assimilation while feeding on virus‐infected plants (Wang *et al*., 2012). Electrical penetration graph analysis of whitefly feeding behaviors demonstrates that virus‐infected whiteflies display impaired feeding on healthy plants. By contrast, when viruliferous whiteflies feed on virus‐infected plants, their feeding is enhanced as evidenced by rapid and effective sap ingestion (He *et al*., 2015). These observations indicate that the beneficial effect of the virus on whiteflies is indirect, and virus‐induced enhancement of whitefly performance is achieved via their shared host plants.

By integrating genetics, genomics, transcriptomics and metabolomics, the multifaceted mechanisms of the pathogenicity factor βC1 action has been revealed. The first discovery was that βC1 encoded by TYLCCNV’s satellite interferes with the plant’s JA pathway to suppress terpenoid synthesis (Zhang *et al*., 2012; Luan *et al*., 2013b). Further studies have shown that βC1 disrupts JA‐regulated plant immunity using three distinct mechanisms. First, by interacting with the plant transcription factor MYC2, βC1 interferes with the plant’s JA‐signaling pathway to reduce downstream terpenoid synthesis (Li *et al*., 2014). Second, βC1 interacts with tobacco protein S‐phase kinase‐associated protein 1, resulting in enhanced JAZ1 stability and the attenuation of the plant’s JA defense responses (Zou *et al*., 2020). Finally, within the phloem, βC1 binds to the plant transcription factor WRKY20. This viral hijacking of WRKY20 causes a spatiotemporal redeployment of plant chemical immunity. Indolic glucosinolates are reduced within the phloem, resulting in beneficial effects for the virus and its whitefly vector. In addition, the increases in methionine‐derived glucosinolates within the leaf lamina negatively impact the performance of the cotton bollworm (a nonvector competitor) (Zhao *et al*., 2019). Further experiments have shown that red light promotes the manipulation of whitefly–plant interactions by TYLCCNV βC1 via stabilization of βC1, which interacts with several PHYTOCHROME‐INTERACTING FACTOR (PIF) transcription factors to compromise terpene synthesis (Zhao *et al*., 2021). These potent and multifaceted impacts of βC1 on plant immunity assures viral manipulation of the JA‐signaling pathway and, by extension, vector success.

### 3. Effects of virus infection on plants: an evolutionary outcome?

The effects of virus infection on plant–insect interactions are often termed ‘virus manipulation’, as virus infection alters many plant traits, including those involved in plant–insect interactions, and some of these traits favor virus transmission (Mauck *et al*., 2012, 2018; Zhang *et al*., 2017; Carr *et al*., 2018; Eigenbrode *et al*., 2018). However, as the most successful kind of organisms on earth, as judged by biomass, plants are not passive bystanders. Instead, plants have developed sophisticated systems, such as hormone‐regulated immune‐signaling networks, to regulate their own response to viruses and their vectors (Pieterse *et al*., 2012). Therefore, should the effects of virus infection on plant–insect interactions be attributed to virus manipulation, or should they instead be interpreted as a result of long‐term adaption of the plants to viruses?

Defense responses to microbial pathogens, such as viruses or phytophagous insects, requires the activation of defense phytohormone pathways triggering the expression of numerous genes, and requires a reallocation of carbon and nitrogen resources to defense. As these resources are often limited for plants in agricultural ecosystems, the metabolic restructuring often leads to suppression of plant growth in most, if not all, cases via the crosstalk between defense and growth‐related phytohormone pathways (Pieterse *et al*., 2012; Vos *et al*., 2013; Kliebenstein, 2016). Upon attack, the deployment of this sophisticated regulatory system to balance growth and reproduction by plants often has ecological costs. For example, to minimize the resources channeled to defense, the resistance traits induced in response to one organism may suppress resistance to another attacker (Vos *et al*., 2013). Indeed, the tradeoff between pathogen and herbivore resistance was observed two decades ago (Felton & Korth, 2000). In the context of tripartite interactions, the reallocation of resources to viral defense equates to reduced resources for defense against insect herbivores and the biosynthesis of nutritional compounds. Collectively, these dynamic and finely tuned changes in gene expression and metabolism may profoundly impact plant–insect interactions. There is now substantial evidence that crosstalk between defense pathways, such as JA–SA antagonism, as well as the integration with daily circadian rhythms, may be a plausible mechanism responsible for the reallocation of resources (Felton & Korth, 2000; Vos *et al*., 2013; Zhou *et al*., 2015).

### 4. Transmission or infection: virus evolution at a glance

As biotrophic pathogens, plant viruses need to overcome host defenses to achieve successful infection. For most plant viruses, their dispersal and spread to new hosts rely on their herbivorous insect vectors (Hogenhout *et al*., 2008; Lefeuvre *et al*., 2019). Plants often use different and antagonistic signaling pathways for defense against viruses and their insect vectors, with the SA‐signaling pathway active against viruses and the JA‐signaling pathway active against insect vectors (Felton & Korth, 2000; Wu & Baldwin, 2010; Pieterse *et al*., 2012; Alazem & Lin, 2015). Hence, nonpersistently transmitted viruses, which rely on short‐term feeding and rapid dispersal of insect vectors for transmission, may achieve productive transmission and infection by suppressing SA signaling and activating JA signaling. However, an obvious conflict arises for persistently transmitted viruses, the transmission of which relies on long‐term feeding of insect vectors on infected plants. Should these viruses manipulate their host plants to maximize infection, which can be achieved via suppression of the SA‐signaling pathway and activation of the JA‐signaling pathway? Alternatively, should these viruses maximize transmission by enhancing vector performance, which can be achieved via activation of the SA‐signaling pathway and suppression of the JA‐signaling pathway?

As summarized by Mauck *et al*. (2012, 2018) and Eigenbrode *et al*. (2018), most studies focusing on persistently transmitted viruses indicate that these viruses manipulate plants to prioritize transmission over infection. For other biotrophic pathogens, such as many plant pathogenic fungi and bacteria, interference with phytohormone‐signaling pathways to favor their own infection is usually prioritized (Kazan & Lyons, 2014). One explanation for the difference in the strategies deployed by plant viruses and other biotrophic pathogens could be the smaller genome size of viruses, which limits their ability to manipulate plants in favor of their own infection. However, this hypothesis is challenged by the ability of geminiviruses to extensively manipulate plant defense responses despite their minute genomes (ranging from 2.5 to 5.2 kb) that encode merely five to seven proteins (Hanley‐Bowdoin *et al*., 2013).

It should always be kept in mind that any successful virus life cycle must balance productive infection and efficient transmission. Strategically, viruses need to ensure that a plant host will support a substantial virus load and yet survive long enough for transmission to occur. Under this scenario, natural selection may favor and promote the persistence of plant viruses that can manipulate their host plants in ways conducive to their own transmission to new host plants. In this context, transmissibility is one of the most important facets for the success of plant viruses, as only the viruses that are transmittable can survive, spread extensively and cause epidemics.

## VI. Prospects

Review of the literature indicates that while significant advances have been made in the last decade, we are just beginning to realize the complexity and dynamics of the modulation of plant–insect interactions by viruses. Of particular relevance to this review and future research endeavors are modulations of plant–insect interactions by viruses through their impact on the synthesis and functioning of phytohormones.

### 1. Role of auxin, GA, CK, BR and ABA

Auxin, GA, CK, BR and ABA concentrations are altered by virus infection (Fraser & Whenham, 1982; Jameson & Clarke, 2002; Benjamins & Scheres, 2008; Pieterse *et al*., 2012; Alazem & Lin, 2017). But their roles in modulating subsequent plant–insect interactions and the outcomes of tripartite interactions have yet to be revealed. With multidisciplinary approaches, this research area is poised for major research advances.

### 2. Effects of genetic factors

Owing to evolutionary pressures, the genotypes of viruses, their insect vectors and shared host plants are continually evolving. Hence, variation occurs between and within species or genotypic variants within a species. This genetic variation in the three partner organisms and their associated morphological and biochemical traits constitute a collection of intrinsic factors that can profoundly influence the outcome of each tripartite interaction. Additionally, morphological, physiological and biochemical traits of an organism are dynamic and may vary with its development/growth stage. For example, Wang *et al*. (2012) found that the assimilation of nutrients from host plants by whiteflies is enhanced during TYLCCNV infection and this benefit is most prominent in young virus‐infected plants. This research area is largely unexplored and will be a fertile ground for new discoveries. Future studies should carefully document the genotypes of each tripartite partner, as well as their developmental stage. This will ensure that these genetic factors are controlled, which should promote an accurate assessment of viral infection on phytohormones and plant–insect interactions and provide a meaningful basis for comparisons between different tripartite systems.

Two other facets of tripartite interactions should also be emphasized in the future. First, some insects can vector plant viruses with different modes of transmission, and their ramifications on phytohormones and tripartite interactions is ready for investigation. For example, whiteflies of the *B. tabaci* species complex transmit begomoviruses in a persistent‐circulative manner and criniviruses in a semipersistent manner (Navas‐Castillo *et al*., 2011; Fiallo‐Olivé *et al*., 2020). While holding the host plant and whitefly species constant, comparisons of plant responses to persistent and semipersistent viruses can be made. These studies should reveal new insights into the orchestration of virus‐induced changes in phytohormones and plant defense traits that determine the outcomes of tripartite interactions. Finally, the sequence of biotic attacker establishment on host plants (e.g. virus first or vector first) influences the outcome of tripartite interactions (P. Li *et al*., 2017) and this research area needs further investigation. This temporal variation in biotic challenges may influence the quantitative and qualitative changes in host plant traits to significantly alter insect vector success, as well as viral acquisition and transmission.

### 3. Effects of environmental factors

Environmental factors include biotic factors interacting directly/indirectly with any of the players of a tripartite system, as well as abiotic factors influencing the interaction network. Major biotic factors include traits of other organisms living on the same plant. In the field, colocalization of multiple insect pests (some as vectors, others as nonvectors) or multiple viruses is common. The dynamics of coinfecting viruses within a host plant and the effect of mixed virus infection on plant–insect interactions may differ significantly from those of single infections. For example, Peñaflor *et al*. (2016) showed that infection of soybeans with bean pod mottle virus increases the palatability of the host plant for its beetle vector to enhance vector acquisition of the virus. However, dual infection with bean pod mottle virus and soybean mosaic virus does not alter host plant palatability. The molecular mechanisms that determine these dynamic differences in plant–insect interactions are yet to be determined.

Viruses may also interact with other pests including nonvector insects. For example, Zhao *et al*. (2019) recently reported that a begomovirus reprograms plant immunity not only to enhance the fitness of its whitefly vector but also to suppress the performance of nonvector insects. As described earlier, this strategy depends on the ability of the viral βC1 protein to disrupt WRKY20 action, resulting in differences in the spatial distribution of indole‐ and methionine‐derived aliphatic glucosinolates. Additional considerations include the organisms at the third trophic level. Natural enemies of the insect vectors may be significantly influenced by viral infection in the tripartite interactions and these natural enemies may influence both vector and nonvector insects.

Environmental abiotic factors include mainly temperature, light, water availability and nutrition/soil. Recently, red light has been shown to be essential for a positive effect of virus infection on the whitefly vector to occur (Zhao *et al*., 2021). Other environmental factors may also significantly modulate plant–virus/plant–insect interactions, thereby impacting the interaction network in each tripartite system. For example, high temperature modulates plant–virus interaction by inhibiting the hypersensitive response induced by viral proteins of potato virus X in *N. benthamiana* plants (Wang *et al*., 2009). In addition, exposure of field‐grown soybean to solar UV‐B radiation increases isoflavonoid content in pods, which is positively correlated with resistance to stink bugs (Zavala *et al*., 2015). Furthermore, abiotic factors often function in combination, adding additional complexity. For example, resistance of 11 accessions of two *Capsicum* species to three species of thrips is found to vary significantly among locations of the field that differed in climate and soil conditions (Visschers *et al*., 2019). These findings highlight the potentially significant impact of environmental abiotic factors on tripartite interactions, and further indicate the need for considerations of climate change and geographic variation in future studies in this respect. However, to date, investigation of the combined effects of abiotic factors on the outcomes of tripartite interactions is lacking. In addition, with the rise of organic agricultural practices, comparisons of traditional and ‘green’ agricultural practices and their impacts on the molecular processes affecting tripartite interactions is an area that is ready for significant advances (Blundell *et al*., 2020).

### 4. New opportunities in unraveling underlying mechanisms

A multidisciplinary and integrated approach to investigating tripartite interactions, as discussed earlier, has become more fruitful as new technologies and model study systems become available. Notably, the exploration of molecular mechanisms from the plant side may benefit from the genetic resources and short life cycle associated with model plants, such as Arabidopsis. In addition, the application of mathematical modeling, as seen in Donnelly *et al*. (2019), will help to garner further insights into patterns of interactions, particularly at the population and ecosystem levels. Development of new technologies now offers further opportunities. For example, genome‐editing techniques enable targeted genetic changes in genomes to impact gene function and epigenetic regulation (Andriy *et al*., 2020). Application of these technologies to any or all of the partners in a tripartite interaction will enable rapid and precise exploration of molecular mechanisms dictating the outcomes of plant–insect–virus interactions, even in nonmodel organisms.

### 5. Field investigations

From an evolutionary and ecological perspective, the importance of investigating tripartite interactions in the field is obvious. Field experiments regarding the effects of virus infection on phytohormones and plant–insect interactions are rare. This is due in part to the fact that these interactions are affected by so many genetic and environmental factors and, therefore, are challenging to analyze quantitatively. As noted earlier, in the field, virus–plant–insect vector interactions are seldom found as isolated systems. Therefore, the outcomes of the tripartite interaction are likely to be substantially modified by other biotic/abiotic factors. Few studies have yet to broach these complex systems. One example is the ‘real world’ studies of Mauck *et al*. (2015), wherein the preference of nonvector herbivores and predators for virus‐infected and uninfected plants in a weedy field setting was investigated. A large number of field studies and their coherent analyses are likely to promote understanding of the ecological/evolutionary significance of the effects of virus infection on phytohormones and plant–insect interactions that extend well beyond tripartite interactions.

## References

[nph17261-bib-0001] Abe H , Ohnishi J , Narusaka M , Seo S , Narusaka Y , Tsuda S , Kobayashi M . 2008. Function of jasmonate in response and tolerance of *Arabidopsis* to thrip feeding. Plant and Cell Physiology 49: 68–80. 18045812 10.1093/pcp/pcm168

[nph17261-bib-0002] Abe H , Tomitaka Y , Shimoda T , Seo S , Sakurai T , Kugimiya S , Tsuda S , Kobayashi M . 2012. Antagonistic plant defense system regulated by phytohormones assists interactions among vector insect, thrips and a tospovirus. Plant and Cell Physiology 53: 204–212. 22180600 10.1093/pcp/pcr173

[nph17261-bib-0003] Akhtar SS , Mekureyaw MF , Pandey C , Roitsch T . 2020. Role of cytokinins for interactions of plants with microbial pathogens and pest insects. Frontiers in Plant Science 10: 1777. 32140160 10.3389/fpls.2019.01777 PMC7042306

[nph17261-bib-0004] Alazem M , Lin KY , Lin NS . 2014. The abscisic acid pathway has multifaceted effects on the accumulation of *Bamboo mosaic virus* . Molecular Plant–Microbe Interactions 27: 177–189. 24224533 10.1094/MPMI-08-13-0216-R

[nph17261-bib-0005] Alazem M , Lin NS . 2015. Roles of plant hormones in the regulation of host‐virus interactions. Molecular Plant Pathology 16: 529–540. 25220680 10.1111/mpp.12204 PMC6638471

[nph17261-bib-0006] Alazem M , Lin NS . 2017. Antiviral roles of abscisic acid in plants. Frontiers in Plant Science 8: 1760. 29075279 10.3389/fpls.2017.01760 PMC5641568

[nph17261-bib-0007] Anderson PK , Cunningham AA , Patel NG , Morales FJ , Epstein PR , Daszak P . 2004. Emerging infectious diseases of plants: pathogen pollution, climate change and agrotechnology drivers. Trends in Ecology and Evolution 19: 535–544. 16701319 10.1016/j.tree.2004.07.021

[nph17261-bib-0008] Andriy B , Daniel G , John L . 2020. Emerging genome engineering tools in crop research and breeding. Methods in Molecular Biology 2072: 165–181. 31541446 10.1007/978-1-4939-9865-4_14

[nph17261-bib-0009] Awmack CS , Leather SR . 2002. Host plant quality and fecundity in herbivorous insects. Annual Review of Entomology 47: 817–844. 10.1146/annurev.ento.47.091201.145300 11729092

[nph17261-bib-0010] Bak A , Patton MF , Perilla‐Henao LM , Aegerter BJ , Casteel CL . 2019. Ethylene signaling mediates potyvirus spread by aphid vectors. Oecologia 190: 139–148. 31065807 10.1007/s00442-019-04405-0

[nph17261-bib-0011] Baliji S , Lacatus G , Sunter G . 2010. The interaction between geminivirus pathogenicity proteins and adenosine kinase leads to increased expression of primary cytokinin‐responsive genes. Virology 402: 238–247. 20399479 10.1016/j.virol.2010.03.023 PMC2876732

[nph17261-bib-0012] Belliure B , Janssen A , Sabelis MW . 2008. Herbivore benefits from vectoring plant virus through reduction of period of vulnerability to predation. Oecologia 156: 797–806. 18392858 10.1007/s00442-008-1027-9 PMC2469278

[nph17261-bib-0013] Benjamins R , Scheres B . 2008. Auxin: the looping star in plant development. Annual Review of Plant Biology 59: 443–465. 10.1146/annurev.arplant.58.032806.103805 18444904

[nph17261-bib-0014] Bernays EA . 2009. Phytophagous insects. In: Vincent R , Carde R , eds. Encyclopedia of insects, 2 ^nd^ edn. New York, NY, USA: Academic Press, 798–800.

[nph17261-bib-0015] Bi H , Fan W , Zhang P . 2017. C4 protein of *Sweet potato leaf curl virus* regulates brassinosteroid signaling pathway through interaction with AtBIN2 and affects male fertility in *Arabidopsis* . Frontiers in Plant Science 8: 1689. 29021807 10.3389/fpls.2017.01689 PMC5623726

[nph17261-bib-0016] Blundell R , Schmidt JE , Igwe A , Cheung AL , Vannette RL , Gaudin ACM , Casteel CL . 2020. Organic management promotes natural pest control through altered plant resistance to insects. Nature Plants 6: 483–491. 32415295 10.1038/s41477-020-0656-9

[nph17261-bib-0017] Boquel S , Giordanengo P , Ameline A . 2011. Divergent effects of PVY‐infected potato plant on aphids. European Journal of Plant Pathology 129: 507–510.

[nph17261-bib-0018] Bouzroud S , Gouiaa S , Hu N , Bernadac A , Mila I , Bendaou N , Smouni A , Bouzayen M , Zouine M . 2018. Auxin response factors (ARFs) are potential mediators of auxin action in tomato response to biotic and abiotic stress (*Solanum lycopersicum*). PLoS ONE 13: e0193517. 29489914 10.1371/journal.pone.0193517 PMC5831009

[nph17261-bib-0019] Broekgaarden C , Caarls L , Vos IA , Pieterse CM , Van Wees SC . 2015. Ethylene: traffic controller on hormonal crossroads to defense. Plant Physiology 169: 2371–2379. 26482888 10.1104/pp.15.01020 PMC4677896

[nph17261-bib-0020] Browse J . 2009. Jasmonate passes muster: a receptor and targets for the defense hormone. Annual Review of Plant Biology 60: 183–205. 10.1146/annurev.arplant.043008.092007 19025383

[nph17261-bib-0021] Carr JP , Donnelly R , Tungadi T , Murphy AM , Jiang SJ , Bravo‐Cazar A , Yoon J , Cunniffe NJ , Glover BJ , Gilligan CA . 2018. Viral manipulation of plant stress responses and host interactions with insects. Advances in Virus Research 102: 177–197. 30266173 10.1016/bs.aivir.2018.06.004

[nph17261-bib-0022] Casteel CL , De Alwis M , Bak A , Dong H , Whitham SA , Jander G . 2015. Disruption of ethylene responses by *Turnip mosaic virus* mediates suppression of plant defense against the green peach aphid vector. Plant Physiology 169: 209–218. 26091820 10.1104/pp.15.00332 PMC4577379

[nph17261-bib-0023] Casteel CL , Yang C , Nanduri AC , De Jong HN , Whitham SA , Jander G . 2014. The NIa‐Pro protein of *Turnip mosaic virus* improves growth and reproduction of the aphid vector, *Myzus persicae* (green peach aphid). The Plant Journal 77: 653–663. 24372679 10.1111/tpj.12417

[nph17261-bib-0024] Castle SJ , Mowry TM , Berger PH . 1998. Differential settling by *Myzus persicae* (Homoptera: Aphididae) on various virus infected host plants. Annals of the Entomological Society of America 91: 661–667.

[nph17261-bib-0025] Chisholm PJ , Sertuvalkul N , Casteel CL , Crowder DW . 2018. Reciprocal plant‐mediated interactions between a virus and a non‐vector herbivore. Ecology 99: 2139–2144. 29999522 10.1002/ecy.2449

[nph17261-bib-0026] Clarke SF , McKenzie MJ , Burritt DJ , Guy PL , Jameson PE . 1999. Influence of *White clover mosaic potexvirus* infection on the endogenous cytokinin content of bean. Plant Physiology 120: 547–552. 10364406 10.1104/pp.120.2.547 PMC59293

[nph17261-bib-0027] Cui HY , Sun YC , Chen FJ , Zhang YJ , Ge F . 2016. Elevated O_3_ and TYLCV infection reduce the suitability of tomato as a host for the whitefly *Bemisia tabaci* . International Journal of Molecular Sciences 17: 1964. 10.3390/ijms17121964 PMC5187764 27916792

[nph17261-bib-0028] De Barro PJ , Liu SS , Boykin LM , Dinsdale AB . 2011. *Bemisia tabaci*: a statement of species status. Annual Review of Entomology 56: 1–19. 10.1146/annurev-ento-112408-085504 20690829

[nph17261-bib-0029] Divi UK , Krishna P . 2009. Brassinosteroid: a biotechnological target for enhancing crop yield and stress tolerance. New Biotechnology 26: 131–136. 19631770 10.1016/j.nbt.2009.07.006

[nph17261-bib-0030] Donnelly R , Cunniffe NJ , Carr JP , Gilligan CA . 2019. Pathogenic modification of plants enhances long‐distance dispersal of non‐persistently transmitted viruses to new hosts. Ecology 100: e02725. 30980528 10.1002/ecy.2725 PMC6619343

[nph17261-bib-0031] Donnelly R , Gilligan CA . 2020. What is pathogen‐mediated insect superabundance? Journal of the Royal Society Interface 17: 20200229. 10.1098/rsif.2020.0229 PMC7536056 32900300

[nph17261-bib-0032] Eigenbrode SD , Bosquepérez NA , Davis TS . 2018. Insect‐borne plant pathogens and their vectors: ecology, evolution, and complex interactions. Annual Review of Entomology 63: 169–191. 10.1146/annurev-ento-020117-043119 28968147

[nph17261-bib-0033] Eigenbrode SD , Ding HJ , Shiel P , Berger PH . 2002. Volatiles from potato plants infected with *Potato leafroll virus attract* and arrest the virus vector, *Myzus persicae* (Homoptera: Aphididae). Proceedings of the Royal Society B: Biological Sciences 269: 455–460. 10.1098/rspb.2001.1909 PMC1690919 11886636

[nph17261-bib-0034] Felton GW , Korth KL . 2000. Trade‐offs between pathogen and herbivore resistance. Current Opinion in Plant Biology 3: 309–314. 10873851 10.1016/s1369-5266(00)00086-8

[nph17261-bib-0035] Fiallo‐Olivé E , Pan LL , Liu SS , Navas‐Castillo J . 2020. Transmission of begomoviruses and other whitefly‐borne viruses: dependence on the vector species. Phytopathology 110: 10–17. 31544592 10.1094/PHYTO-07-19-0273-FI

[nph17261-bib-0036] Fiebig M , Poehling HM , Borgemeister C . 2004. *Barley yellow dwarf virus*, wheat, and *Sitobion avenae*: a case of trilateral interactions. Entomologia Experimentalis et Applicata 110: 11–21.

[nph17261-bib-0037] Fraser RSS , Whenham RJ . 1982. Plant growth regulators and virus infection: a critical review. Plant Growth Regulation 1: 37–59.

[nph17261-bib-0038] Gadhave KR , Dutta B , Coolong T , Srinivasan R . 2019. A non‐persistent aphid‐transmitted *Potyvirus* differentially alters the vector and non‐vector biology through host plant quality manipulation. Scientific Reports 9: 2503. 30792431 10.1038/s41598-019-39256-5 PMC6385306

[nph17261-bib-0039] Gangwere SK . 2008. Food habits of insects. In: Cpinera JL , ed. Encyclopedia of Entomology. New York, NY, USA: Springer, 1504–1512.

[nph17261-bib-0040] Groen SC , Sanjie J , Murphy AM , Cunniffe NJ , Westwood JH , Davey MP , Bruce TJA , Caulfield JC , Furzer OJ , Reed A *et al*. 2016. Virus infection of plants alters pollinator preference: a payback for susceptible hosts? PLoS Pathogens 12: e1005790. 27513727 10.1371/journal.ppat.1005790 PMC4981420

[nph17261-bib-0041] Guilfoyle TJ , Hagen G . 2007. Auxin response factors. Current Opinion in Plant Biology 10: 453–460. 17900969 10.1016/j.pbi.2007.08.014

[nph17261-bib-0042] Hanley‐Bowdoin L , Bejarano ER , Robertson D , Mansoor S . 2013. Geminiviruses: masters at redirecting and reprogramming plant processes. Nature Reviews Microbiology 11: 777–788. 24100361 10.1038/nrmicro3117

[nph17261-bib-0043] He WB , Li J , Liu SS . 2015. Differential profiles of direct and indirect modification of vector feeding behaviour by a plant virus. Scientific Reports 5: 7682. 25567524 10.1038/srep07682 PMC4286760

[nph17261-bib-0044] He XC , Xu HX , Gao GC , Zhou XJ , Zheng XS , Sun YJ , Yang YJ , Tian JC , Lu ZX . 2014. Virus‐mediated chemical changes in rice plants impact the relationship between non‐vector planthopper *nilaparvata lugens* stål and its egg parasitoid *Anagrus nilaparvatae* Pang et Wang. PLoS ONE 9: e105373. 25141278 10.1371/journal.pone.0105373 PMC4139343

[nph17261-bib-0045] He YZ , Wang YM , Yin TY , Fiallo‐Olivé E , Liu YQ , Hanley‐Bowdoin L , Wang XW . 2020. A plant DNA virus replicates in the salivary glands of its insect vector via recruitment of host DNA synthesis machinery. Proceedings of the National Academy of Sciences, USA 117: 16928–16937. 10.1073/pnas.1820132117 PMC7382290 32636269

[nph17261-bib-0046] Hogenhout SA , Ammar ED , Whitfield AE , Redinbaugh MG . 2008. Insect vector interactions with persistently transmitted viruses. Annual Review of Phytopathology 46: 327–359. 10.1146/annurev.phyto.022508.092135 18680428

[nph17261-bib-0047] Jameson PE , Clarke SF . 2002. Hormone‐virus interactions in plants. Critical Reviews in Plant Sciences 21: 205–228.

[nph17261-bib-0048] Jiménez‐Martínez ES , Bosque‐Pérez NA , Berger PR , Ding HJ . 2004. Volatile cues influence the response of *Rhopalosiphum padi* (Homoptera: Aphididae) to *Barley yellow dwarf virus*‐infected transgenic and untransformed wheat. Environmental Entomology 33: 1207–1216.

[nph17261-bib-0049] Jiu M , Zhou XP , Tong L , Xu J , Yang X , Wan FH , Liu SS . 2007. Vector‐virus mutualism accelerates population increase of an invasive whitefly. PLoS ONE 2: e182. 17264884 10.1371/journal.pone.0000182 PMC1773017

[nph17261-bib-0050] Joffrey M , Chesnais Q , Spicher F , Verrier E , Ameline A , Couty A . 2018. Plant virus infection influences bottom‐up regulation of a plant‐aphid‐parasitoid system. Journal of Pest Science 91: 361–372.

[nph17261-bib-0051] Kanakala S , Ghanim M . 2019. Global genetic diversity and geographical distribution of *Bemisia tabaci* and its bacterial endosymbionts. PLoS ONE 14: e0213946. 30889213 10.1371/journal.pone.0213946 PMC6424426

[nph17261-bib-0052] Kazan K , Lyons R . 2014. Intervention of phytohormone pathways by pathogen effectors. The Plant Cell 26: 2285–2309. 24920334 10.1105/tpc.114.125419 PMC4114936

[nph17261-bib-0053] Kazan K , Manners JM . 2009. Linking development to defense: auxin in plant‐pathogen interactions. Trends in Plant Science 14: 373–382. 19559643 10.1016/j.tplants.2009.04.005

[nph17261-bib-0054] Kersch‐Becker MF , Thaler JS . 2014. Virus strains differentially induce plant susceptibility to aphid vectors and chewing herbivores. Oecologia 174: 883–892. 24178835 10.1007/s00442-013-2812-7

[nph17261-bib-0055] Kliebenstein . 2016. False idolatry of the mythical growth versus immunity tradeoff in molecular systems plant pathology. Physiological and Molecular Plant Pathology 95: 55–59.

[nph17261-bib-0056] Laney AG , Chen PY , Korth KL . 2018. Interactive effects of aphid feeding and virus infection on host gene expression and volatile compounds in salt‐stressed soybean, *Glycine max* (L.) Merr. Arthropod‐Plant Interactions 12: 401–413.

[nph17261-bib-0057] Lefeuvre P , Martin DP , Elena SF , Shepherd DN , Roumagnac P , Varsani A . 2019. Evolution and ecology of plant viruses. Nature Review Microbiology 17: 632–644. 31312033 10.1038/s41579-019-0232-3

[nph17261-bib-0058] Leng P , Ji Q , Asp T , Frei UK , Ingvardsen CR , Xing Y , Studer B , Redinbaugh M , Jones M , Gajjar P *et al*. 2017. Auxin binding protein 1 reinforces resistance to *Sugarcane mosaic virus* in maize. Molecular Plant 10: 1357–1360. 28827193 10.1016/j.molp.2017.07.013

[nph17261-bib-0059] Leon‐Reyes A , Spoel SH , De Lange ES , Abe H , Kobayashi M , Tsuda S , Millenaar FF , Welschen RAM , Ritsema T , Pieterse CMJ . 2009. Ethylene modulates the role of NONEXPRESSOR OF PATHOGENESIS‐RELATED GENES1 in cross talk between salicylate and jasmonate signaling. Plant Physiology 149: 1797–1809. 19176718 10.1104/pp.108.133926 PMC2663751

[nph17261-bib-0060] Lewsey MG , Murphy AM , Maclean D , Dalchau N , Westwood JH , Macaulay K , Bennett MH , Moulin M , Hanke DE , Powell G *et al*. 2010. Disruption of two defensive signaling pathways by a viral RNA silencing suppressor. Molecular Plant–Microbe Interactions 23: 835–845. 20521947 10.1094/MPMI-23-7-0835

[nph17261-bib-0061] Li JY , Li CY , Smith SM . 2017. Hormone metabolism and signaling in plants. Cambridge, UK: Academic Press.

[nph17261-bib-0062] Li P , Liu C , Deng WH , Yao DM , Pan LL , Li YQ , Liu YQ , Liang Y , Zhou XP , Wang XW . 2019. Plant begomoviruses subvert ubiquitination to suppress plant defenses against insect vectors. PLoS Pathogens 15: e1007607. 30789967 10.1371/journal.ppat.1007607 PMC6400417

[nph17261-bib-0063] Li P , Shu YN , Fu S , Liu YQ , Zhou XP , Liu SS , Wang XW . 2017. Vector and non‐vector insect feeding reduces subsequent plant susceptibility to virus transmission. New Phytologist 215: 699–710. 10.1111/nph.14550 28382644

[nph17261-bib-0064] Li R , Weldegergis BT , Li J , Jung C , Qu J , Sun Y , Tee C , van Loon JJA , Dicke M , Chua NH *et al*. 2014. Virulence factors of geminivirus interact with MYC2 to subvert plant resistance and promote vector performance. The Plant Cell 26: 4991–5008. 25490915 10.1105/tpc.114.133181 PMC4311212

[nph17261-bib-0065] Li Y , Yang Y , Hu Y , Liu H , He M , Yang Z , Kong F , Liu X , Hou X . 2019. DELLA and EDS1 form a feedback regulatory module to fine‐tune plant growth‐defense tradeoff in Arabidopsis. Molecular Plant 12: 1485–1498. 31382023 10.1016/j.molp.2019.07.006

[nph17261-bib-0066] Liu BM , Preisser EL , Shi XB , Wu HT , Li CY , Xie W , Wang XL , Wu QJ , Zhang YJ . 2017. Plant defense negates pathogen manipulation of vector behaviour. Functional Ecology 31: 1574–1581.

[nph17261-bib-0067] Liu SS , Colvin J , De Barro PJ . 2012. Species concepts as applied to the whitefly *Bemisia tabaci* systematics: how many species are there? Journal of Integrate Agriculture 11: 176–186.

[nph17261-bib-0068] Liu WY , Chiou SJ , Ko CY , Lin TY . 2011. Functional characterization of three ethylene response factor genes from *Bupleurum kaoi* indicates that *BkERFs* mediate resistance to *Botrytis cinerea* . Journal of Plant Physiology 168: 375–381. 20728241 10.1016/j.jplph.2010.07.006

[nph17261-bib-0069] Liu X , He YY , Xie W , Wu QJ , Zhang YJ , Liu Y , Wang SL . 2018. Infection of tomato by *Tomato yellow leaf curl virus* alters the foraging behaviour and parasitism of the parasitoid, *Encarsia formosa* on *Bemisia tabaci* . Journal of Asia‐Pacific Entomology 21: 548–552.

[nph17261-bib-0070] Lu J , Ju H , Zhou G , Zhu C , Erb M , Wang X , Wang P , Lou YG . 2011. An EAR motif‐containing ERF transcription factor affects herbivore‐induced signaling, defense and resistance in rice. The Plant Journal 68: 583–596. 21831212 10.1111/j.1365-313X.2011.04709.x

[nph17261-bib-0071] Luan JB , Wang YL , Wang J , Wang XW , Liu SS . 2013a. Detoxification activity and energy cost is attenuated in the whiteflies feeding on begomovirus‐infected tobacco plants. Insect Molecular Biology 22: 597–607. 23889516 10.1111/imb.12048

[nph17261-bib-0072] Luan JB , Yao DM , Zhang T , Walling LL , Yang M , Wang YJ , Liu SS . 2013b. Suppression of terpenoid synthesis in plants by a virus promotes its mutualism with vectors. Ecology Letters 16: 390–398. 23279824 10.1111/ele.12055

[nph17261-bib-0073] Mach J . 2012. Why wiry? Tomato mutants reveal connections among small RNAs, auxin response factors, virus infection, and leaf morphology. The Plant Cell 24: 3486. 23001038 10.1105/tpc.112.240911 PMC3480282

[nph17261-bib-0074] Malka O , Santos‐Garcia D , Feldmesser E , Sharon E , Krause‐Sakate R , Delatte H , van Brunschot S , Patel M , Visendi P , Mugerwa H *et al*. 2018. Species‐complex diversification and host‐plant associations in *Bemisia tabaci*: a plant‐defense, detoxification perspective revealed by RNA‐Seq analyses. Molecular Ecology 27: 4241–4256. 30222226 10.1111/mec.14865 PMC6334513

[nph17261-bib-0075] Mandadi KK , Scholthof KBG . 2013. Plant immune responses against viruses: how does a virus cause disease? The Plant Cell 25: 1489–1505. 23709626 10.1105/tpc.113.111658 PMC3694688

[nph17261-bib-0076] Mansour A , Al‐Musa A . 1992. *Tomato yellow leaf curl virus*: host range and virus‐vector relationships. Plant Pathology 41: 122–125.

[nph17261-bib-0077] Maramorosch K . 1957. Reversal of virus‐caused stunting in plants by gibberellic acid. Science 126: 651–652. 17836627 10.1126/science.126.3275.651

[nph17261-bib-0078] Martins DDS , Ventura JA , Lima RDCA , Culik MP , Costa H , Ferreira PSF . 2012. Interaction between papaya meleira virus (PMeV) infection of papaya plants and Mediterranean fruit fly infestation of fruits. Crop Protection 36: 7–10.

[nph17261-bib-0079] Mauck KE , Bosque‐Pérez NA , Eigenbrode SD , De Moraes CM , Mescher MC . 2012. Transmission mechanisms shape pathogen effects on host‐vector interactions: evidence from plant viruses. Functional Ecology 26: 1162–1175.

[nph17261-bib-0080] Mauck KE , Chesnais Q , Shapiro LR . 2018. Evolutionary determinants of host and vector manipulation by plant viruses. Advances in Virus Research 101: 189–250. 29908590 10.1016/bs.aivir.2018.02.007

[nph17261-bib-0081] Mauck KE , De Moraes CM , Mescher MC . 2010. Deceptive chemical signals induced by a plant virus attract insect vectors to inferior hosts. Proceeding of the National Academy of Science, USA 107: 3600–3605. 10.1073/pnas.0907191107 PMC2840436 20133719

[nph17261-bib-0082] Mauck KE , De Moraes CM , Mescher MC . 2014. Biochemical and physiological mechanisms underlying effects of cucumber mosaic virus on host‐plant traits that mediate transmission by aphid vectors. Plant, Cell & Environment 37: 1427–1439. 10.1111/pce.12249 24329574

[nph17261-bib-0083] Mauck KE , Smyers E , De Moraes CM , Mescher MC . 2015. Virus infection influences host plant interactions with non‐vector herbivores and predators. Functional Ecology 29: 662–673.

[nph17261-bib-0084] McGrath KC , Dombrecht B , Manners JM , Schenk PM , Edgar CI , Maclean DJ , Scheible WR , Udvardi MK , Kazan K . 2005. Repressor‐ and activator‐type ethylene response factors functioning in jasmonate signaling and disease resistance identified via a genome‐wide screen of *Arabidopsis* transcription factor gene expression. Plant Physiology 139: 949–959. 16183832 10.1104/pp.105.068544 PMC1256008

[nph17261-bib-0085] McMenemy LS , Hartley SE , Macfarlane SA , Karley AJ , Shepherd T , Johnson SN . 2012. Raspberry viruses manipulate the behaviour of their insect vectors. Entomologia Experimentalis et Applicata 144: 56–68.

[nph17261-bib-0086] Medina‐Ortega KJ , Bosque‐Pérez NA , Ngumbi E , Jiménez‐Martínez ES , Eigenbrode SD . 2009. *Rhopalosiphum padi* (Hemiptera: Aphididae) responses to volatile cues from *Barley yellow dwarf virus*‐infected wheat. Environmental Entomology 38: 836–845. 19508794 10.1603/022.038.0337

[nph17261-bib-0087] Miyaji T , Yamagami A , Kume N , Sakuta M , Osada H , Asami T , Arimoto Y , Nakano T . 2014. Brassinosteroid‐related transcription factor BIL1/BZR1 increases plant resistance to insect feeding. Bioscience, Biotechnology, and Biochemistry 78: 960–968. 10.1080/09168451.2014.910093 25036120

[nph17261-bib-0088] van Molken TV , de Caluwe H , Hordijk CA , Leon‐Reyes A , Snoeren TAL , van Dam NM , Stuefer JF . 2012. Virus infection decreases the attractiveness of white clover plants for a non‐vectoring herbivore. Oecologia 170: 433–444. 22526939 10.1007/s00442-012-2322-z PMC3439618

[nph17261-bib-0089] Mur LAJ , Kenton P , Atzorn R , Miersch O , Wasternack C . 2006. The outcomes of concentration‐specific interactions between salicylate and jasmonate signaling include synergy, antagonism, and oxidative stress leading to cell death. Plant Physiology 140: 249–262. 16377744 10.1104/pp.105.072348 PMC1326048

[nph17261-bib-0090] Nachappa P , Margolies DC , Nechols JR , Whitfield AE , Rotenberg D . 2013. *Tomato spotted wilt virus* benefits a non‐vector arthropod, *Tetranychus urticae*, by modulating different plant responses in tomato. PLoS ONE 8: e75909. 24058708 10.1371/journal.pone.0075909 PMC3776767

[nph17261-bib-0091] Navarro L , Bari R , Achard P , Lison P , Nemri A , Harberd NP , Jones JD . 2008. DELLAs control plant immune responses by modulating the balance of jasmonic acid and salicylic acid signaling. Current Biology 18: 650–655. 18450451 10.1016/j.cub.2008.03.060

[nph17261-bib-0092] Navas‐Castillo J , Fiallo‐Olivé E , Sánchez‐Campos S . 2011. Emerging virus diseases transmitted by whiteflies. Annual Review of Phytopathology 49: 219–248. 10.1146/annurev-phyto-072910-095235 21568700

[nph17261-bib-0093] Ngumbi E , Eigenbrode SD , Bosquepérez NA , Ding H , Rodriguez A . 2007. *Myzus persicae* is arrested more by blends than by individual compounds elevated in headspace of PLRV‐infected potato. Journal of Chemical Ecology 33: 1733–1747. 17680312 10.1007/s10886-007-9340-z

[nph17261-bib-0094] Nolan T , Chen J , Yin Y . 2017. Cross‐talk of brassinosteroid signaling in controlling growth and stress responses. Biochemical Journal 474: 2641–2661. 10.1042/BCJ20160633 PMC6296487 28751549

[nph17261-bib-0095] Paponov IA , Dindas J , Krol E , Friz T , Budnyk V , Teale W *et al*. 2019. Auxin‐induced plasma membrane depolarization is regulated by auxin transport and not by AUXIN BINDING PROTEIN 1. Frontiers in Plant Science 9: 1953. 30705682 10.3389/fpls.2018.01953 PMC6344447

[nph17261-bib-0096] Patton MF , Bak A , Sayre JM , Heck ML , Casteel CL . 2019. A polerovirus, *Potato leafroll virus*, alters plant‐vector interactions using three viral proteins. Plant, Cell & Environment 43: 387–399. 10.1111/pce.13684 31758809

[nph17261-bib-0097] Peñaflor MFGV , Mauck KE , Alves KJ , De Moraes CM , Mescher MC . 2016. Effects of single and mixed infections of *Bean pod mottle virus* and *Soybean mosaic virus* on host‐plant chemistry and host‐vector interactions. Functional Ecology 30: 1648–1659.

[nph17261-bib-0098] Pieterse CM , Van der Does D , Zamioudis C , Leon‐Reyes A , Van Wees SC . 2012. Hormonal modulation of plant immunity. Annual Review of Cell and Developmental Biology 28: 489–521. 10.1146/annurev-cellbio-092910-154055 22559264

[nph17261-bib-0099] Preston CA , Lewandowski C , Alexander JE , Baldwin IT . 1999. *Tobacco mosaic virus* inoculation inhibits wound‐induced jasmonic acid‐mediated responses within but not between plants. Planta 209: 87–95. 10467034 10.1007/s004250050609

[nph17261-bib-0100] Prince DC , Drurey C , Zipfel C , Hogenhout SA . 2014. The leucine‐rich repeat receptor‐like kinase BRASSINOSTEROID INSENSITIVE1‐ASSOCIATED KINASE1 and the cytochrome P450 PHYTOALEXIN DEFICIENT3 contribute to innate immunity to aphids in Arabidopsis. Plant Physiology 164: 2207–2219. 24586042 10.1104/pp.114.235598 PMC3982773

[nph17261-bib-0101] Robert‐Seilaniantz A , Navarro L , Bari R , Jones JD . 2007. Pathological hormone imbalances. Current Opinion in Plant Biology 10: 372–379. 17646123 10.1016/j.pbi.2007.06.003

[nph17261-bib-0102] Satoh K , Shimizu T , Kondoh H , Hiraguri A , Sasaya T , Choi IR , Omura T , Kikuchi S . 2011. Relationship between symptoms and gene expression induced by the infection of three strains of *Rice dwarf virus* . PLoS ONE 6: e18094. 21445363 10.1371/journal.pone.0018094 PMC3062569

[nph17261-bib-0103] Scholthof KBG , Adkins S , Czosnek H , Palukaitis P , Jacquot E , Hohn T , Hohn B , Sauder S , Candresse T , Ahlquist P *et al*. 2011. Top 10 plant viruses in molecular plant pathology. Molecular Plant Pathology 12: 938–954. 22017770 10.1111/j.1364-3703.2011.00752.x PMC6640423

[nph17261-bib-0104] Shi XB , Yang G , Yan S , Xin T , Zhou XG , Zhang DY , Liu Y . 2016. Aphid performance changes with plant defense mediated by *Cucumber mosaic virus* titer. Virology Journal 13: 70. 27103351 10.1186/s12985-016-0524-4 PMC4840961

[nph17261-bib-0105] Stout MJ , Thaler JS , Thomma BPHJ . 2006. Plant‐mediated interactions between pathogenic microorganisms and herbivorous arthropods. Annual Review of Entomology 51: 663–689. 10.1146/annurev.ento.51.110104.151117 16332227

[nph17261-bib-0106] Su Q , Mescher MC , Wang SL , Chen G , Xie W , Wu QJ , Wang WK , Zhang YJ . 2016. *Tomato yellow leaf curl virus* differentially influences plant defense responses to a vector and a non‐vector herbivore. Plant, Cell & Environment 39: 597–607. 10.1111/pce.12650 26436779

[nph17261-bib-0107] Su Q , Preisser EL , Zhou XM , Xie W , Liu BM , Wang SL , Wu QJ , Zhang YJ . 2015. Manipulation of host quality and defense by a plant virus improves performance of whitefly vectors. Journal of Economic Entomology 108: 11–19. 26470098 10.1093/jee/tou012

[nph17261-bib-0108] Sun YC , Pan LL , Ying FZ , Li P , Wang XW , Liu SS . 2017. Jasmonic acid‐related resistance in tomato mediates interactions between whitefly and whitefly transmitted virus. Scientific Reports 7: 566. 28373670 10.1038/s41598-017-00692-w PMC5428805

[nph17261-bib-0109] Tack AJM , Dicke M . 2013. Plant pathogens structure arthropod communities across multiple spatial and temporal scales. Functional Ecology 27: 633–645.

[nph17261-bib-0110] Tao T , Zhou CJ , Wang Q , Chen XR , Sun Q , Zhao TY , Ye JC , Wang Y , Zhang ZY , Zhang YL *et al*. 2017. *Rice black streaked dwarf virus* P7–2 forms a SCF complex through binding to *Oryza sativa* SKP1‐like proteins, and interacts with GID2 involved in the gibberellin pathway. PLoS ONE 12: e0177518. 28494021 10.1371/journal.pone.0177518 PMC5426791

[nph17261-bib-0111] Thaler JS , Agrawal AA , Halitschke R . 2010. Salicylate‐mediated interactions between pathogens and herbivores. Ecology 91: 1075–1082. 20462121 10.1890/08-2347.1

[nph17261-bib-0112] Tomitaka Y , Abe H , Sakurai T , Tsuda S . 2015. Preference of the vector thrips *Frankliniella occidentalis* for plants infected with thrips‐non‐transmissible *Tomato spotted wilt virus* . Journal of Applied Entomology 139: 250–259.

[nph17261-bib-0113] Ton J , Flors V , Mauch‐Mani B . 2009. The multifaceted role of ABA in disease resistance. Trends in Plant Science 14: 310–317. 19443266 10.1016/j.tplants.2009.03.006

[nph17261-bib-0114] Tungadi T , Groen SC , Murphy AM , Pate AE , Iqbal J , Bruce TJA , Cunniffe NJ , Carr JP . 2017. *Cucumber mosaic virus* and its 2b protein alter emission of host volatile organic compounds but not aphid vector settling in tobacco. Virology Journal 14: 91. 28468686 10.1186/s12985-017-0754-0 PMC5415739

[nph17261-bib-0115] Ueda H , Kugimiya S , Tabata J , Kitamoto H , Mitsuhara I . 2019. Accumulation of salicylic acid in tomato plant under biological stress affects oviposition preference of *Bemisia. tabaci* . Journal of Plant Interactions 14: 73–78.

[nph17261-bib-0116] Verma V , Ravindran P , Kumar PP . 2016. Plant hormone‐mediated regulation of stress responses. BMC Plant Biology 16: 86. 27079791 10.1186/s12870-016-0771-y PMC4831116

[nph17261-bib-0117] Visschers IGS , Peters JL , Timmermans LLH , Edwards E , Ferrater JB , Balatero CH , Stratongjun M , Bleeker PM , van Herwijnen Z , Glawe GA *et al*. 2019. Resistance to three thrips species in *Capsicum* spp. depends on site conditions and geographic regions. Journal of Applied Entomology 00: 1–13.

[nph17261-bib-0118] Vitti A , Nuzzaci M , Scopa A , Tataranni G , Remans T , Vangronsveld J , Sofo A . 2013. Auxin and cytokinin metabolism and root morphological modifications in *Arabidopsis thaliana* seedlings infected with cucumber mosaic virus (CMV) or exposed to cadmium. International Journal of Molecular Science 14: 6889–6902. 10.3390/ijms14046889 PMC3645669 23531542

[nph17261-bib-0119] Vos IA , Pieterse CMJ , Van Wees SCM . 2013. Costs and benefits of hormone‐regulated plant defenses. Plant Pathology 62: 43–55.

[nph17261-bib-0120] de Vos M , Jander G . 2010. Volatile communication in plant‐aphid interactions. Current Opinion in Plant Biology 13: 366–371. 20627668 10.1016/j.pbi.2010.05.001

[nph17261-bib-0121] Wang J , Bing XL , Li M , Ye GY , Liu SS . 2012. Infection of tobacco plants by a begomovirus improves nutritional assimilation by a whitefly. Entomologia Experimentalis et Applicata 144: 191–201.

[nph17261-bib-0122] Wang SM , Hou XL , Ying LI , Cao XW , Zhang S , Wang F . 2011. Effects of Turnip mosaic virus (TuMV) on endogenous hormones and transcriptional level of related genes in infected non‐heading Chinese cabbage. Journal of Nanjing Agricultural University 34: 13–19 [in Chinese with English abstract].

[nph17261-bib-0123] Wang XW , Blanc S . 2021. Insect transmission of plant single‐stranded DNA viruses. Annual Review of Entomology 66: 389–405. 10.1146/annurev-ento-060920-094531 32931313

[nph17261-bib-0124] Wang Y , Bao Z , Zhu Y , Hua J . 2009. Analysis of temperature modulation of plant defense against biotrophic microbes. Molecular Plant–Microbe Interactions 22: 498–506. 19348568 10.1094/MPMI-22-5-0498

[nph17261-bib-0125] Wang YM , He YZ , Ye XT , He WZ , Liu SS , Wang XW . 2020. Whitefly HES1 binds to the intergenic region of Tomato yellow leaf curl China virus and promotes viral gene transcription. Virology 542: 54–62. 32056668 10.1016/j.virol.2020.01.009 PMC7031692

[nph17261-bib-0126] Westwood JH , Groen SC , Du ZY , Murphy AM , Anggoro DT , Tungadi T , Luang‐In V , Lewsey MG , Rossiter JT , Powell G *et al*. 2013a. A trio of viral proteins tunes aphid‐plant interactions in *Arabidopsis thaliana* . PLoS ONE 8: e83066. 24349433 10.1371/journal.pone.0083066 PMC3859657

[nph17261-bib-0127] Westwood JH , McCann L , Naishi M , Dixon H , Murphy AM , Stancombe MA , Bennett MH , Powell G , Webb AAR , Carr JP . 2013b. A viral RNA silencing suppressor interferes with abscisic acid‐mediated signalling and induces drought tolerance in *Arabidopsis thaliana* . Molecular Plant Pathology 14: 158–170. 23083401 10.1111/j.1364-3703.2012.00840.x PMC6638696

[nph17261-bib-0128] Whenham RJ , Fraser RSS , Brown LP , Payne JA . 1986. *Tobacco‐mosaic‐virus*‐induced increase in abscisic‐acid concentration in tobacco leaves. Planta 168: 592–598. 24232338 10.1007/BF00392281

[nph17261-bib-0129] Wu D , Qi T , Li WX , Tian H , Gao H , Wang JJ , Ge J , Yao RF , Ren CM , Wang XB *et al*. 2017. Viral effector protein manipulates host hormone signaling to attract insect vectors. Cell Research 27: 402–415. 28059067 10.1038/cr.2017.2 PMC5339842

[nph17261-bib-0130] Wu J , Baldwin IT . 2010. New insights into plant responses to the attack from insect herbivores. Annual Review of Genetics 44: 1–24. 10.1146/annurev-genet-102209-163500 20649414

[nph17261-bib-0146] Wu XJ , Xu S , Zhao PZ , Zhang X , Yao XM , Sun YW , Fang RX , Ye J . 2019. The Orthotospovirus nonstructural protein NSs suppresses plant MYC‐regulated jasmonate signaling leading to enhanced vector attraction and performance. PLoS Pathogens 15: e1007897. 31206553 10.1371/journal.ppat.1007897 PMC6598649

[nph17261-bib-0131] Wu XJ , Ye J . 2020. Manipulation of jasmonate signaling by plant viruses and their insect vectors. Viruses 12: 148. 10.3390/v12020148 PMC7077190 32012772

[nph17261-bib-0132] Xie K , Li L , Zhang H , Wang R , Tan X , He Y , Hong G , Li J , Ming F , Yao X *et al*. 2018. Abscisic acid negatively modulates plant defense against *Rice black‐streaked dwarf virus* infection by suppressing the jasmonate pathway and regulating ros levels in rice. Plant, Cell & Environment. 41: 2504–2514. 10.1111/pce.13372 29920686

[nph17261-bib-0133] Yang JY , Iwasaki M , Machida C , Machida Y , Zhou X , Chua NH . 2008. betaC1, the pathogenicity factor of TYLCCNV, interacts with AS1 to alter leaf development and suppress selective jasmonic acid responses. Genes and Development 22: 2564–2577. 18794352 10.1101/gad.1682208 PMC2546693

[nph17261-bib-0134] Yifhar T , Pekker I , Peled D , Friedlander G , Pistunov A , Sabban M , Wachsman G , Alvarez JP , Amsellem Z , Eshed Y . 2012. Failure of the tomato trans‐acting short interfering RNA program to regulate AUXIN RESPONSE FACTOR3 and ARF4 underlies the wiry leaf syndrome. The Plant Cell 24: 3575–3589. 23001036 10.1105/tpc.112.100222 PMC3480288

[nph17261-bib-0135] Yu MH , Zhao ZZ , He JX . 2018. Brassinosteroid signaling in plant‐microbe interactions. International Journal of Molecular Sciences 19: 4091. 10.3390/ijms19124091 PMC6320871 30563020

[nph17261-bib-0136] Zavala JA , Mazza CA , Dillon FM , Chludil HD , Ballaré CL . 2015. Soybean resistance to stink bugs (*Nezara viridula* and *Piezodorus guildinii*) increases with exposure to solar UV‐B radiation and correlates with isoflavonoid content in pods under field conditions. Plant, Cell & Environment 38: 920–928. 10.1111/pce.12368 24811566

[nph17261-bib-0137] Zerbini FM , Briddon RW , Idris A , Martin DP , Moriones E , Navas‐Castillo J , Rivera‐Bustamante R , Roumagnac P , Varsani A , ICTV Report Consortium . 2017. ICTV virus taxonomy profile: *Geminiviridae* . Journal of General Virology 98: 131–133. 10.1099/jgv.0.000738 PMC5802298 28284245

[nph17261-bib-0138] Zhang L , Zhang F , Melotto M , Yao J , He SY . 2017. Jasmonate signaling and manipulation by pathogens and insects. Journal of Experimental Botany 68: 1371–1385. 28069779 10.1093/jxb/erw478 PMC6075518

[nph17261-bib-0139] Zhang T , Luan JB , Qi JF , Huang CJ , Li M , Zhou XP , Liu SS . 2012. Begomovirus‐whitefly mutualism is achieved through repression of plant defenses by a virus pathogenicity factor. Molecular Ecology 21: 1294–1304. 22269032 10.1111/j.1365-294X.2012.05457.x

[nph17261-bib-0140] Zhao PZ , Yao XM , Cai CX , Li R , Du J , Sun YW , Wang MY , Zou Z , Wang QM , Kliebenstein DJ *et al*. 2019. Viruses mobilize plant immunity to deter nonvector insect herbivores. Science Advances 5: eaav9801. 31457079 10.1126/sciadv.aav9801 PMC6703867

[nph17261-bib-0141] Zhao PZ , Zhang X , Gong YQ , Wang D , Xu DQ , Wang N , Sun YW , Gao LB , Liu SS , Deng XW *et al*. 2021. Red‐light is an environmental effector for mutualism between begomovirus and its vector whitefly. PLoS Pathogens 17: e1008770. 33428670 10.1371/journal.ppat.1008770 PMC7822537

[nph17261-bib-0142] Zhou M , Wang W , Karapetyan S , Mwimba M , Marques J , Buchler NE , Dong X . 2015. Redox rhythm reinforces the circadian clock to gate immune response. Nature 523: 472–476. 26098366 10.1038/nature14449 PMC4526266

[nph17261-bib-0143] Zhu S , Gao F , Cao X , Chen M , Ye G , Wei C , Li Y . 2005. The *Rice dwarf virus* P2 protein interacts with ent‐kaurene oxidases *in vivo*, leading to reduced biosynthesis of gibberellins and rice dwarf symptoms. Plant Physiology 139: 1935–1945. 16299167 10.1104/pp.105.072306 PMC1310571

[nph17261-bib-0144] Ziebell H , Murphy AM , Groen SC , Tungadi T , Westwood JH , Lewsey MG , Moulin M , Kleczkowski A , Smith AG , Stevens M *et al*. 2011. *Cucumber mosaic virus* and its 2b RNA silencing suppressor modify plant–aphid interactions in tobacco. Scientific Reports 1: 187. 22355702 10.1038/srep00187 PMC3240964

[nph17261-bib-0145] Zou C , Shu YN , Yang JJ , Pan LL , Zhao J , Chen N , Liu SS , Wang XW . 2020. Begomovirus virulence factor βC1 attenuates tobacco defense to whitefly via interacting with plant SKP1. Frontiers in Plant Science 11: 574557. 32973859 10.3389/fpls.2020.574557 PMC7481519

